# Effect of *GSTM2-5* polymorphisms in relation to tobacco smoke exposures on lung function growth: a birth cohort study

**DOI:** 10.1186/1471-2466-13-56

**Published:** 2013-09-03

**Authors:** Melannie Alexander, Wilfried Karmaus, John W Holloway, Hongmei Zhang, Graham Roberts, Ramesh J Kurukulaaratchy, Syed Hasan Arshad, Susan Ewart

**Affiliations:** 1 Department of Epidemiology and Biostatistics, University of South Carolina, Columbia, USA; 2 Division of Epidemiology, Biostatistics and Environmental Health, School of Public Health, University of Memphis, 236A Robison Hall, Memphis, TN 38152, USA; 3 Human Genetics and Medical Genomics, Human Development & Health, Faculty of Medicine, University of Southampton, Southampton, UK; 4 Clinical and Experimental Sciences, Faculty of Medicine, University of Southampton, Southampton, UK; 5 David Hide Asthma and Allergy Research Centre, Isle of Wight, UK; 6 College of Veterinary Medicine, Michigan State University, East Lansing, MI, USA

**Keywords:** Smoking, Lung function, Diplotype, Human, Longitudinal study, Epigenetics, Methylation quantitative trait loci

## Abstract

**Background:**

Genetic variation within *GSTM2-5* genes may interfere with detoxification of environmental compounds, thereby having a detrimental effect on lung function following exposures such as tobacco smoke. We aim to investigate the influence of variants and associated methylation in the *GSTM* gene cluster with changes in lung function growth during adolescence.

**Methods:**

Growth in forced expiratory volume (FEV_1_), forced vital capacity (FVC), and change in FEV_1_/FVC ratio measures were obtained from children in the Isle of Wight birth cohort at ages 10 and 18. Illumina GoldenGate assays were used to genotype 10 tagging polymorphisms from *GSTM2* (rs574344 and rs12024479), *GSTM3* (rs1537236, rs7483, and rs10735234), *GSTM4* (rs668413, rs560018, and rs506008), and *GSTM5* (rs929166 and rs11807) genes. Diplotypes were generated in the software Phase 3.0.2. DNA methylation was measured in over 450,000 CpG sites using the Infinium HumanMethylation450 BeadChip (Illumina 450K) in a subsample of 245 18-year olds from the Isle of Wight birth cohort. Gender, age, *in utero* smoke exposure, secondhand smoke exposure (SHS), and current smoking status were assessed via questionnaire; smoke exposures were validated with urine cotinine. We used linear mixed models to estimate the effect of *GSTM* diplotypes on lung function across time and examine interactions with tobacco smoke.

**Results:**

1,121 (77%) out of 1,456 children had information on lung function at ages 10 or 18. After adjustment for false discovery rate, one diplotype in *GSTM3* had a detrimental effect on changes in FEV_1_ (p=0.03), and another diplotype in *GSTM3* reduced FVC (p=0.02) over time. No significant interactions with smoking were identified. SHS significantly modified the relationship between diplotypes and methylation levels in one *GSTM2* CpG site; however, this site did not predict lung function outcomes at age 18. Joint effects of *GSTM* loci and CpG sites located within these loci on adolescent lung growth were detected.

**Conclusions:**

Diplotypes within *GSTM2-5* genes are associated with lung function growth across adolescence, but do not appear to modify the effect of tobacco smoke exposures on adolescent lung growth. Interactions between DNA methylation and diplotypes should be taken into account to gain further understanding on lung function in adolescence.

## Background

Pregnancy is a critical period of lung growth and development, making the fetus susceptible to environmental exposures [1]. Indeed, *in utero* exposure to tobacco smoke has shown detrimental effects on lung health [1-4]. *In utero* tobacco smoke exposure may affect morphogenesis and maturation of the lungs, leading to impaired lung health by adulthood [2]. The toxic effect of tobacco smoke exposure might be reduced through metabolizing xenobiotic products, thus minimizing oxidative stress, a physiological event caused by the imbalance between reactive oxygen species and the body’s ability to detoxify these products [5]. However, if detoxification is inefficient or absent within the mother or the child due to genetic polymorphisms, the exposed child may have a decreased ability to remove reactive oxygen species properly. Furthermore, effects of prenatal and early childhood exposure to tobacco smoke may persist through time, thus leading to reduced lung function growth even in adolescence. Additional exposures during adolescence, such as personal smoking, may further affect the growth trajectory in lung function in vulnerable individuals as well; therefore, it is important to control for these factors as well in order to determine the pure joint effects of tobacco smoke exposures and genetic polymorphisms responsible for detoxification.

The glutathione S-transferases (GST) are a superfamily of catalytic proteins responsible for detoxification of xenobiotic compounds, in conjugation with glutathione. There are seven classes of the GST superfamily: alpha, mu, pi, sigma, theta, omega, and zeta [5]. While the effects of glutathione S-transferase mu 1 (*GSTM1*) deletion have been extensively studied, results of studies examining these effects on various pulmonary outcomes are in conflict [6-11]. For instance, findings in a study among German schoolchildren aged 9 to 11 years only found a significant interaction between *GSTM1* status and *in utero* smoke exposure on maximum mid expiratory flow (MMEF) levels [10], however Henderson et al. while confirming a detrimental effect of intrauterine tobacco smoke exposure on childhood lung function found no strong evidence of modification by maternal or child *GSTM1* genotype [12]. Regarding growth, only one study found an association between *GSTM1* gene function and lung function growth, where children with the *GSTM1* deletion had slower lung function growth compared to those with the normal genotype [6]. In addition to copy number variation of the *GSTM1*gene, genetic variation in the form of single nucleotide polymorphisms (SNPs) within other members of the GST family also needs to be considered as it may lead to reduced expression of enzymes that detoxify harmful products that impact lung function growth.

One explanation for conflicting results of studies of the *GSTM1* deletion is the failure to consider the impact of SNP variation in the adjacent *GSTM2-5* loci. This is highlighted by the study of Breton *et al*. where *GSTM2* was associated with growth in FEV_1_ and MMEF; *GSTM4* was associated with FEV_1_, FVC, and MMEF; *GSTM3* and *GSTM5* were associated with MMEF; and *in utero* smoke exposure modified the effect of *GSTM2* haplotypes on growth in FEV_1_ and FVC [13]. Because of these independent effects of variation within the *GSTM2-5* loci on lung health, it is important to take this into account when examining the effect of *GSTM1* variation on lung function growth in adolescence.

An alternative explanation for variation between studies in genetic epidemiology is variation in environmental exposure between cohorts [14]. Effects of genetic polymorphisms may be only seen in exposed populations, or vice versa, and in some circumstances, ‘flip-flop’ effects may be present where the effect of alleles on disease outcome is reversed depending on environmental exposure. Environmental exposure may also result in epigenetic effects such as altered DNA methylation that may either mask or synergize with the effects of germline genetic variation on phenotype [15]. For example *in utero* tobacco smoke exposure has been shown to result in changes in DNA methylation in cord blood DNA [16] that persist until adulthood (unpublished observations).

Thus far, only one study has reported the effects of genetic variation found in *GSTM2-5* and their interaction effect with tobacco smoke exposure on lung function levels and growth in late adolescence, where *in utero* smoke exposure modified the effect of *GSTM2* on lung function growth [13]. Since active smoking only had marginally significant effects on FEV_1_ and FEV_1_/FVC at age 18 in the Isle of Wight (IOW) cohort (unpublished observations), the effect may only be detected in those with a genetic susceptibility, acting through an epigenetic mechanism. To expand these findings, we analyzed data collected in the IOW birth cohort study. Specifically, we explore the interactive relationships between smoking status and individual *GSTM* diplotypes. Furthermore, we validated whether or not smoke exposures modified the effect of diplotypes on methylation levels of CpG sites within this gene cluster and determined whether or not methylation levels of these CpG sites affected lung function levels at age 18.

## Methods

### Study population

From January 1989 to February 1990 in the IOW, UK, 1,536 children were born; 1,456 mother-child pairs were enrolled into the cohort study. The local research ethics committee (National Research Ethics Service, NRES Committee South Central–Southampton B) approved the study and informed written parental consent was obtained for all participants at recruitment and subsequently at each follow-up. The IOW birth cohort has been described in detail elsewhere [17-19].

### Lung function measurement

At ages 10 and 18 years, pulmonary function tests, which included forced expiratory volume in 1 second (FEV_1_), forced vital capacity (FVC), and FEV_1_/FVC ratio, were performed on all consenting children (n=1,121). Because mixed models can handle unbalanced data sets, characteristics of the analytic sample (children with pulmonary function test data available at age 10, age 18, or both) were compared with characteristics of the total cohort. Details of lung function measurement performed at ages 10 and 18 years were reported elsewhere [19]. Briefly, lung function measurements were conducted using a Koko Spirometer and software with a desktop portable device (PDS Instrumentation, Louisville, USA), according to American Thoracic Society guidelines [20]. Children were required to be free from respiratory infection for 14 days and were not taking oral steroids. In addition, they abstained from any β agonist medication for six hours and caffeine intake for at least four hours.

### Laboratory analysis

Details on genomic DNA extraction from whole blood samples collected at age 18 and genotyping can be found in the online supplement (Additional file 1). Genomic DNA obtained from a subsample of female subjects (N=245) were bisulfite-converted and applied to the Infinium HumanMethylation450 BeadChip from Illumina (Illumina, San Diego, CA, USA), which is used to assess methylation in over 484,000 CpG sites found within approximately 24,000 genes. Arrays were processed using a standard protocol as described elsewhere [21], with multiple identical control samples assigned to each bisulphite conversion batch to assess assay variability and samples randomly distributed on microarrays to control against batch effects. The BeadChips were scanned using a BeadStation, and the methylation level (beta value) calculated for each queried CpG locus using the Methylation Module of BeadStudio software. All *GSTM2-5* SNPs of interest were checked against a database containing SNPs that may artificially alter *GSTM2-5* CpG site methylation produced from dbSNP build v130 [22]. Also, CpG sites were excluded from further analyses if nearby probe SNPs affected their methylation.

### Covariates

Smoking during pregnancy was ascertained at enrollment, soon after the birth of the child. Information on tobacco smoking by mothers, by fathers or any other individual within the household was recorded at recruitment and updated at each follow-up at ages 1, 2, 4, 10, and 18 to assess secondhand smoke (SHS) exposure for ages 10 and 18 years. Personal smoking status of the offspring was collected via questionnaires and validated through urinary cotinine levels (ng/mmol) at age 18. Height in centimeters (cm) and weight in kilograms (kg) were assessed by study investigators at ages 1, 2, 4, 10, and 18 years; body mass index (BMI) was calculated. To ensure that effects of *GSTM2-5* loci were independent of *GSTM1*, rs366631, a pseudo-SNP that acts as a *GSTM1* deletion marker, was included as one of the covariates [23].

### Statistical analysis

Haploview 4.2 [24] was used to assess each SNP for Hardy-Weinberg equilibrium (HWE) and create linkage disequilibrium (LD) blocks using the Gabriel *et al.* method.[25] SNPs with a HWE p-value ≥ 0.01 and with a minor allele frequency (MAF) ≥ 5% were considered for diplotype formation. Haplotypes were generated in Phase 2.1 [26,27] and were linked as pairs if the probability of constituting a pair was >0.60. The advantage of using diplotypes is that each study subject has only one diplotype. Usage of haplotypes would double the sample size, thereby deflating variance and p-values [28]. Diplotypes with frequencies of 5% or less were combined into a “minor pairs” category. Diplotypes were characterized according to the sequence of the alleles involved (e.g. TC_TC or both haplotypes having thymidine and cytosine).

Statistical analyses were conducted using SAS 9.2 (Cary, NC) to assess effect of SNPs and diplotypes on lung function growth. Linear mixed models were applied to adjust for correlations between the repeated measurements. Models were built with an unstructured covariance matrix, which requires the least amount of constraints. Other covariance matrices were applied, but no other covariance matrix improved the fit (Akaike information criteria and Bayesian Schwarz information criterion).

**Figure 1:**
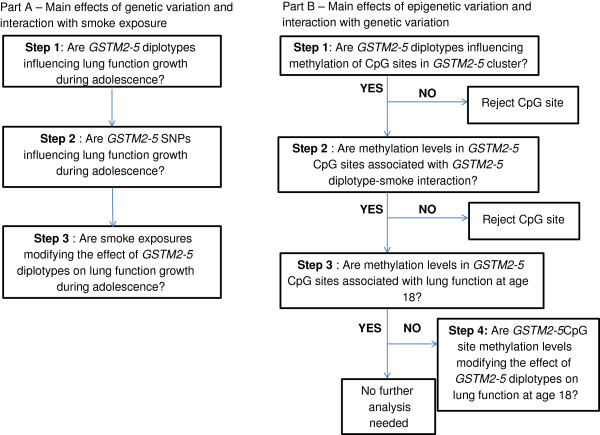
**Overview of data analysis plan.** Part A describes analysis plan to assess main genetic effects of *GSTM2-5* diplotypes and single nucleotide polymorphisms (SNPs) and their interactions with smoke exposures. Part B outlines the CpG site screening process and the plan to find methylation quantitative trait loci (methQTL), conditioned on smoking, and the joint effect of modifiable genetic variants (modGVs) and DNA methylation on lung function.

Separate models for changes of FEV_1_, FVC, and FEV_1_/FVC over time were created. Significance of interactions (α = 0.05) was assessed first, then confounders were removed if they did not change the estimates of diplotype categories by at least 10%. The most frequent diplotype served as the reference group, allowing us to assess the effects of less common pairs on lung function outcomes. Due to the number of hypothesis tests being conducted, diplotypes were declared significant at a false discovery rate (FDR) of 0.05 within each lung function outcome. The overall effect of the diplotype blocks were evaluated through the global F-test. To determine joint effects of diplotypes and smoke exposures, separate models were constructed for each type of diplotype-smoking interaction and were examined for significance through the global F-test. Part A of Figure 1 provides an overview of the analysis plan. To validate self-reported responses of maternal and active smoking, we compared cotinine levels of individuals belonging to exposed and unexposed groups using the Wilcoxon-Mann–Whitney test. Individuals who did not actively smoke were examined to assess the pure effect of SHS exposure.

Methylation quantitative trait loci (methQTL) analyses were performed via Wilcoxon-Mann–Whitney tests to select CpG sites housed in the *GSTM2-5* cluster that varied by diplotype among a subset of participants with methylation information (n=245). Because tobacco smoke exposure is postulated to interact with certain diplotypes and produce epigenetic changes, general linear models were used to assess the joint effect of diplotypes and tobacco smoke exposure on methylation levels of *GSTM2-5* CpG sites. *GSTM2-5* CpG sites that were the result of a significant interaction were then tested whether they modified the effect of diplotypes (modifiable genetic variants [modGV]) [15] on lung function levels at age 18 using general linear models, adjusting for confounders. Joint effects of these CpG sites and other *GSTM2-5* modGVs were also examined. Part B of Figure 1 gives an overview of this analysis plan. Findings were verified through path analytical models. More details on path analysis are found in the supplement (Additional file 1).

## Results

Data on lung function measures were available for 1,121 children (Table 1). No significant differences were found between this subsample and the total cohort (n = 1,456). All SNPs in the *GSTM2-5* loci were in HWE and had a MAF ≥ 5% (Table 2). SNPs within each gene formed their own blocks based on linkage disequilibrium (LD) measures, where the r^2^ value was 0.8 or greater. A total of four blocks were generated (Additional file 1: Figure S2). The each block was comprised of the following SNPs: *GSTM2* rs574344 and rs12024479; *GSTM3* rs1537236, rs7483, and rs7537234; *GSTM4* rs668413, rs560018, and rs506008; and *GSTM5* rs929166 and rs11807; (Additional file 1: Table S1). All reported p-values were adjusted for multiple testing for an FDR of 0.05, when applicable. Only *GSTM3* and *GSTM5* show direct associations with gain in FEV1 or FVC (Table 3). Statistical analyses of the individual effects of SNPs within the *GSTM2-5* cluster on gain in each lung function outcome yielded no significant findings (Additional file 2: Table S2). When looking at main effects of *GSTM2-5* diplotypes by age, only *GSTM5* diplotypes contributed to FEV_1_ levels at age 10 (Additional file 3: Table S3a). Several interactions between *GSTM2-5* diplotypes and smoke exposures were detected, but only at age 18. SHS modified the relationship between GSTM2 and FEV1 and FVC levels (p_interaction_ = 0.004 and p_interaction_ = 0.0003, respectively) (Additional file 3: Table S4b); SHS modified the relationship between GSTM3 and FEV1 levels (p_interaction_ = 0.04) (Additional file 3: Additional file 3: Table S5b; and active smoking modified the relationship between GSTM5 and FEV1 and FVC levels (p_interaction_ = 0.004 and p_interaction_ = 0.05, respectively) (Additional file 3: Table S7c).

**Table 1:** Characteristics of subsample with lung function measurements and the total Isle of Wight (IOW) cohort

	**Subsample with lung function measurements (n=1121)**		**Total cohort (n=1536)**		
Variables	N	%	N	%	p-value
Gender					
Males	557	49.7	786	51.2	0.45
Females	564	50.3	750	48.8	
Missing	0		0		
*In utero* smoke exposure					
Yes	253	22.7	384	25.2	0.12
No	864	77.4	1137	74.8	
Missing	419		15		
SHS smoke exposure at age 10					
Yes	508	45.3	848	56.7	0.26
No	609	54.3	647	43.3	
Missing	419		41		
SHS smoke exposure at age 18					
Yes	563	55.2	716	56.7	0.49
No	457	44.8	548	43.3	
Missing	516		272		
Active smoking at age 18					
Yes	276	26.9	368	28.8	0.30
No	752	73.2	910	71.2	
Missing	508		258		
	Median (5^th^ percentile, 95^th^ percentile); n				
Height at age 10 (cm)	138.7 (129.1, 149.5); 1026		138.7 (129.1, 149.5); 1043		0.98
Missing	510		493		
Height at age 18 (cm)	171.0 (156.5, 186.5); 918		171.0 (156.5, 187.0); 994		0.64
Missing	618		542		
BMI at age 10 (kg/m^2^)	17.4 (14.7, 24.1); 1026		17.4 (14.7, 23.9); 1043		0.92
Missing	510		493		
BMI at age 18 (kg/m^2^)	22.2 (18.2, 32.2); 896		22.2 (18.2, 32.2); 964		0.91
Missing	640		572		

*SHS* Secondhand smoke, *BMI* Body Mass Index.

**Table 2:** **Genotype information of *GSTM2-5 *single nucleotide polymorphisms**

**Gene**	**SNP**	**Base pair position**	**Location**	**Genotype**	**Genotype frequency (%)**	**Minor allele frequency (MAF) (%)**
*GSTM2*	rs574344	110015037	Intron	AA	0.5	7.6
				AT	14.2	
				TT	85.3	
	rs12024479	110021609	Flanking 3^*’*^-UTR*	CC	27.2	48.1
				CG	49.4	
				GG	23.4	
*GSTM3*	rs1537236	110080495	3^*’*^-UTR*	AA	23.4	49.3
				AG	51.8	
				GG	24.8	
	rs7483	110081224	Exon	AA	9.3	29.7
				AG	40.8	
				GG	50.0	
	rs10735234	110083464	Intron	AA	32.3	34.5
				AG	49.0	
				GG	18.7	
*GSTM4*	rs668413	109997467	Flanking 3^*’*^-UTR*	AA	17.3	41.0
				AC	47.4	
				CC	35.3	
	rs560018	110001883	Intron	AA	42.7	34.7
				AG	45.3	
				GG	12.1	
	rs506008	110003222	Exon	AA	2.1	14.2
				AG	24.2	
				GG	73.7	
*GSTM5*	rs929166	110056697	Intron	AA	55.2	26.7
				AC	36.2	
				CC	8.6	
	rs11807	110062265	3^*’*^-UTR*	AA	66.0	19.0
				AG	30.1	
				GG	3.9	

*Untranslated region.

**Table 3:** **Adjusted linear mixed models examining the main effects of diplotypes within *GSTM2-5 *cluster on repeated lung function measurements from ages 10 to 18 years**

			**Forced expiratory volume in 1 second (FEV**_**1**_**)**		**Forced Vital Capacity (FVC)**		**FEV**_**1**_**/FVC**	
**Gene**	**N**	Diplotype*	**β (mL)**	**p-value§**	**β (mL)**	**p-value§**	**β (%)**	**p-value§**
*GSTM2*	1020	TC_AC	-5.93	0.97	-24.16	0.80	0.69	0.76
		TC_TC	-25.11	0.97	-37.94	0.34	0.14	0.77
		TG_AC	-6.09	0.97	-16.20	0.80	0.38	0.76
		TG_TG	8.69	0.97	-5.15	0.80	0.50	0.76
		Minor diplotypes	5.27	0.97	-40.58	0.80	2.18	0.76
		TC_TG	REF	--	REF	--	REF	--
		p-value‡	0.78		0.59		0.85	
*GSTM3*	1037	AAA_AAA	-47.38	0.11	-50.5538	0.11	0.62	0.73
		AGA_AAA	-56.42	0.09	**-83.1151**	**0.02**	0.74	0. 73
		AGA_GGG	-38.32	0.11	-34.2516	0.15	0.26	0.79
		GGA_GGG	-41.55	0.23	-60.1746	0.11	0.81	0. 73
		GGG_GGG	**-63.81**	**0.03**	-51.9142	0.09	-0.15	0.79
		Minor diplotypes	-53.28	0.09	-54.6330	0.09	-0.17	0.79
		AAA_GGG	REF	--	REF	--	REF	--
		p-value‡	0.12		0.08		0.72	
*GSTM4*	1028	AGG_AGG	-0.37	0.99	28.10	0.42	-0.53	0.60
		CAA_AGG	24.05	0.98	34.38	0.42	-0.10	0.88
		CAG_AAG	-13.55	0.98	-23.75	0.57	0.38	0.79
		CAG_CAA	15.47	0.98	37.69	0.42	-0.51	0.60
		CAG_CAG	-8.92	0.98	12.74	0.57	-0.67	0.60
		Minor diplotypes	6.55	0.98	35.15	0.42	-0.94	0.60
		CAG_AGG	REF	--	REF	--	REF	--
		p-value‡	0.91		0.54		0.69	
*GSTM5*	1002	AA_AG	23.81	0.48	6.13	0.89	0.48	0.67
		AA_CA	**53.26**	**0.04**	31.88	0.50	0.60	0.67
		AG_CA	-24.22	0.48	-30.40	0.50	-0.42	0.67
		CA_CA	-6.40	0.83	4.47	0.89	-0.47	0.67
		Minor diplotypes	-41.33	0.48	-51.04	0.50	-0.27	0.78
		AA_AA	REF	--	REF	--	REF	--
		p-value‡	**0.02**		0.24		0.45	

Note: Bold indicates statistically significant difference in lung function compared to referent group (p ≤ 0.05);

* Comprised of SNPs as described in the Results section; †Models adjusted for sex, rs366631, *in utero* smoke exposure, current smoking, secondhand smoke exposure, and body mass index (BMI); ‡ Global F-test p-value: combined effect of diplotypes on repeated lung function measurements; § After adjustment for false discovery rate (FDR).

### Multivariable analyses

#### ***GSTM2***

Diplotypes within the *GSTM2* locus did not significantly contribute to gain in FEV_1_, FVC, and change in FEV_1_/FVC ratio (Table 3). Additionally, no statistically significant interactions between diplotypes and tobacco smoke exposures were detected (Additional file 3: Tables S8a-c).

#### ***GSTM3***

The overall effect of variations in diplotypes within *GSTM3* neared significance for gain in both FEV_1_ and FVC (p = 0.12 and p = 0.08, respectively). After adjustment for false discovery rate, one diplotype was associated with significantly lower gain in FEV_1_ (GGG_GGG: -63.81 mL, p = 0.03) (Table 3). This diplotype also produced detrimental effects on gain in FVC, but it was not significant (GGG_GGG: -51.91, p = 0.09). An additional diplotype was associated with significantly lower gain in FVC (AGA_AAA: -83.12 mL, p = 0.02). In regard to tobacco smoke interactions, SHS exposure marginally modified the relationship between *GSTM3* diplotypes and change in FEV1/FVC (p_interaction_ = 0.06) (Additional file 3: Table S9b). Statistically significant interactions between diplotypes within *GSTM3* and *in utero* smoke exposure and personal smoking at age 18 were not observed (Additional file 3: Tables S9a and S9c).

#### ***GSTM4***

Overall, diplotypes created in the *GSTM4* locus had no significant effects on gain in FEV_1_, FVC, and change in FEV_1_/FVC ratio (Table 3). Additionally, no significant interactions with *in utero* smoke exposure, SHS exposure, and personal smoking at age 18 were observed (Additional file 3: Tables S10a-c).

#### ***GSTM5***

Global F-tests indicated a significant contribution of *GSTM5* diplotypes to gain in FEV_1_ (p = 0.02, Table 3). Only one pair showed a statistically significant positive relationship with gain in FEV_1_ (AA_CA: 53.26 mL, p = 0.04). Although the interaction between *GSTM5* diplotypes and SHS exposure appeared to affect FEV_1_/FVC change, this relationship was not statistically significant (p = 0.08) (Additional file 3: Table S11b). No other statistically significant interactions were seen between this diplotype group and *in utero* smoke exposure and current smoking (Additional file 3: Tables S11a and S11c).

### Methylation analyses

Although no effects of significant interactions on lung function growth were present in this study, the *GSTM2*×*in utero* smoke exposure interaction was found to be significant in Breton *et al.*[13], suggesting that this smoke exposure changed the function of this gene through epigenetic modifications. As a result, methylation levels in this gene cluster were investigated for their role in lung function. After removal of 25 CpG sites that may be affected by probe SNPs, methQTL analyses revealed that several CpG site methylation levels were dependent on genetic variants of the specific gene: diplotypes of the *GSTM2* diplotypes predicted three CpG site methylation levels; *GSTM3* diplotypes predicted five CpG site methylation levels; *GSTM4* gene predicted five CpG site methylation levels; and *GSTM5* diplotypes predicted one CpG site methylation levels (Additional file 3: Table S12).

When looking at the joint effect of *GSTM2-5* diplotypes and tobacco smoke exposures (SHS and active smoking), only SHS exposure at the age of 18 modified the relationship between *GSTM2* diplotypes and one CpG site found in *GSTM2*: cg06970744 (p_interaction_ = 0.01; Table 4). However, these sites did not predict lung function levels at age 18 (Additional file 3: Table S13). The joint effect of this CpG site and other modGVs had a significant relationship with FEV_1_ (cg06970744× *GSTM5*: p_interaction_ = 0.02) and FVC levels at age 18 (cg06970744× *GSTM5*: p_interaction_ = 0.02) (Table 5). Increasing levels of methylation at cg06970744 produced increased FEV_1_ and FVC levels for individuals with the CA_CA diplotype in *GSTM5*.

**Table 4:** **Adjusted estimates of *GSTM2 *diplotypes on *GSTM2 *CpG site methylation by secondhand smoke exposure status at age 18**

	**cg03942855†**				**cg06970744†**			
	**Not exposed (n = 93)**		**Exposed (n = 130)**		**Not exposed (n = 93)**		**Exposed (n = 130)**	
Diplotype*	**β (%)**	**p-value§**	**β (%)**	**p-value§**	**β (%)**	**p-value§**	**β (%)**	**p-value§**
TC_AC	-2.09	0.31	-1.61	0.33	-0.53	0.80	-2.16	0.34
TC_TC	1.62	0.31	-1.80	0.03	2.19	0.42	-2.75	0.02
TG_AC	-1.92	0.33	-4.12	0.002	2.64	0.51	-3.14	0.07
TG_TG	-1.99	0.31	-1.99	0.02	-2.38	0.42	-3.28	0.01
Minor diplotypes	-3.88	0.33	2.26	0.50	-4.04	0.57	3.23	0.48
TC_TG	REF	--	REF	--	REF	--	REF	--
p_interaction_‡	**0.03**				**0.01**			

* Comprised of SNPs as described in the Results section; †Models adjusted for *in utero* smoke exposure and personal smoking status; ‡ Global F-test p-value: significance of interaction; § After adjustment for false discovery rate (FDR).

**Table 5:** **Adjusted means of FEV**_**1 **_**and FVC in mL by diplotype and CpG methylation percentile**

				**Methylation percentile for cg06970744†,§**			
**Outcome**	**Gene**	**n**	Diplotype*	**25th percentile**	**50th percentile**	**75th percentile**	**p**_**interaction **_**‡**
FEV_1_	*GSTM5*	203	AA_AG	3427.61	3457.13	3497.90	**0.02**
			AA_CA	3584.62	3549.29	3500.49	
			AG_CA	3584.46	3536.99	3471.43	
			CA_CA	3206.02	3349.07	3546.64	
			Minor diplotypes	3527.80	3721.73	3989.59	
			AA_AA	3499.59	3503.89	3509.84	
FVC	*GSTM5*	203	AA_AG	4020.50	4036.67	4059.00	**0.02**
			AA_CA	4091.55	4067.27	4033.75	
			AG_CA	3864.29	3951.14	4071.09	
			CA_CA	3850.02	4016.73	4246.98	
			Minor diplotypes	4004.33	4292.32	4690.09	
			AA_AA	3975.89	3981.64	3989.57	

*Comprised of SNPs as described in the Results section, means for minor diplotypes grouping in *GSTM2* were not estimable; †Models adjusted for rs366631, *in utero* smoke exposure, current smoking, secondhand smoke exposure, body mass index (BMI) and height; ‡ Global F-test p-value: significance of interaction; § Name of methylation site is derived from Illumina ID.

Results of the path analyses revealed no indirect effects of diplotypes on lung function through methylation at cg06970744 at age 18 (Additional file 3: Table S14).

## Discussion

Results suggest that variation within the *GSTM3* and *GSTM5* loci had significant impact on lung function outcomes at age 18 after adjustment for confounding. Some diplotypes, but none of their interactions with smoke exposure (*in utero*, SHS, or active smoking exposure at 18), produced an independent, statistically significant relationship with FEV_1_ and FVC, but not with FEV_1_/FVC. Additional analyses suggest a multi-stage model. First, DNA methylation was affected by diplotypes conditionally on smoking exposure; second, an altered DNA methylation modified the effect of diplotypes on lung function (acting as modGVs), leading to significant effects of *GSTM2* and *GSTM5* on FEV_1_ and FVC. Path analyses show that methylation at cg06970744 did not lie on the pathway between *GSTM5* diplotypes and lung function, further providing evidence that methylation at certain sites can modify the effect of genetic variation on an outcome. Also, methylation at this site appears to be unaffected by nearby SNPs (unpublished observations).

Several *GSTM3* diplotypes had strong, negative effects on gain in lung function. SNPs in *GSTM3* included a functional SNP (rs7483). Previous studies have found an association with this SNP and Alzheimer’s disease [29,30]. Breton *et al.* have previously examined the association of rs7483 with lung function, and similar to the present study, no significant associations were found [13]. *GSTM4* had a synonymous coding SNP (rs506008). This SNP produced positive, but insignificant effects on lung function across time and thus more than likely does not make contributions to lung function at the diplotype level. There are no reported associations between rs506008 and any disease outcomes. Although *GSTM5* had no functional SNPs, there was a significant lung function improvement in those who possessed the AA_CA diplotype, demonstrating a need to investigate this particular region. Only one of the SNPs (rs11807) was previously reported in the literature, showing a strong association with hypertension [31]; however, no association with lung function had been reported.

Due to previous findings that showed lower lung function outcomes due to various tobacco smoke exposures [2-4,32], we assessed the critical period where lung development may be severely impaired by tobacco smoke exposure. No two-way interaction effects of tobacco smoke with *GSTM2-5* loci were seen, which is in contrast to findings by Breton *et al.*, who reported interaction effects with *GSTM2* and *in utero* tobacco smoke [13]. This discrepancy may be either due to misclassification of the exposure and/or insufficient sample size in our study or missing replication in the study by Breton *et al.*[13]. Because questions involving tobacco smoke exposure at age 18 were validated with urinary cotinine levels (Additional file 3: Table S9), there is no suggestion of major misclassification, as those who were active smokers or were exposed to SHS had significantly higher levels of cotinine compared to nonsmokers. The study by Breton *et al.* included at least one more repeated measurement [13], hence, it is possible that the present study does not have equal statistical power to detect interactions. However, there is a need of replication studies to examine the role of these genes on lung function in conjunction with tobacco smoke exposure, especially in different environments. It is also necessary to consider that other environmental and lifestyle exposures including air pollution and paracetamol (acetaminophen) use [33] may also alter oxidative stress [34] and mask or falsely indicate an effect of related exposures. Air pollution was not controlled in the study by Breton *et al.*[13], but was reported to modify the effect of a similarly functioning gene (*GSS*) on lung function growth in another study of this group [33]. Because it has been suggested in the literature that tobacco smoke exposure, especially *in utero*[35], may epigenetically modify these genes, hence changing the function of these genes, we tested the effect of CpG sites on lung function levels at age 18. Although there were no independent effects of these CpG site methylation levels on lung function outcomes, it is interesting to note that these effects were not seen until joint effects of *GSTM2* and *GSTM5* diplotypes were taken into account, indicating that DNA methylation may modify the effect of genetic variants on lung function, a mechanism that needs further investigation [15]. These findings may also help explain the lack of consistency between the present study’s findings and the findings of Breton *et al.*[13]. It may not be the genotype that produces inconsistent results, but rather different DNA methylation levels in different study groups that accounted for the discrepancy.

To further lend validity to this study, selection bias was not apparent regarding availability of lung function data. Approximately 95% (1456/1536) of mother-child pairs were enrolled into this study; and those who underwent pulmonary function tests were not significantly different from the total cohort (Table 1). Also, lung function measurements were obtained under standardized conditions in a prospective manner, decreasing the likelihood of information bias. With respect to genotyping data, all SNPs that were shared with Breton *et al.* were in HWE and had comparable frequencies with Caucasians in their sample [13]. These polymorphisms also agreed substantially in their association with FEV_1_ and FVC with our findings (Additional file 1: Figure S2 and Additional file 2: Figure S3). In regard to haplotypes, diplotypes (haplotype-pairs) were used, thus reducing uncertainty and subsequently misclassification of individuals, which is often encountered in haplotype association studies [36]. Also, our population was homogeneous, meaning controlling for population stratification is unnecessary and ensures HWE, which appeared to be an issue for Breton *et al.*[13], possibly resulting in different findings. While SNPs included in the haplotype analysis of the latter study were in HWE within ethnic groups, they were not in HWE when examining the entire population [13]. This is problematic because inclusion of SNPs that deviate from HWE may produce spurious associations between haplotypes and lung function [37,38]. In addition, the findings by Breton *et al.*[13] may be due to population stratification. The authors identified ancestry indicators, controlled these as confounders but did not stratify their analysis by these markers. Nevertheless, population stratification addresses the possibility that haplotypes may reveal different associations in different ethnic/racial strata. This may also account for the lack of agreement of single SNP effects on lung function outcomes between the present study and the Breton *et al.* study [13].

Despite the strengths of our study, some limitations are present. First, because maternal smoking and active smoking during adolescence is obtained via self-reported questionnaires, misclassification of this exposure is possible. Our previous publications have shown that maternal smoking during pregnancy interacts with the *IL13* and *IL1RN* genes and increases the risk of asthma and wheezing [39,40]. Hence, ascertainment of maternal smoking seemed to provide valid information. However, maternal smoking × *IL13* and *IL1RN* gene interaction assumes an effect of smoking on these genes. Against that, *GSTM2-5* × maternal smoking interactions assume that the genes regulate the toxicity of tobacco smoke, possibly via epigenetic mechanisms. Also, based on results of the methylation analyses in the present study, the joint effect of maternal smoking and *GSTM2-5* diplotypes did not influence methylation levels of CpG sites within the *GSTM2-5* cluster; however, these methylation levels were captured at age 18 and therefore we cannot ascertain whether or not epigenetic changes had taken place due to this particular exposure nor can we determine that methylation levels at age 18 are representative of early-life methylation profiles. Regarding validation of other smoke exposures, passive smoke exposure and active smoking at age 18 was associated with increased cotinine levels. Also, active smoking at age 18 led to deficits in FEV_1_/FVC after adjustments for *GSTM5* diplotype, *GSTM1* genotype, sex, BMI, height, *in utero* smoke exposure, and SHS (Additional file 3: Table S16). Reduced FEV_1_/FVC has previously been observed in adolescent smokers [41].

Second, not all SNPs used in the Breton *et al.* study were genotyped in this work. Therefore combinations of SNPs that were not included in the haplotype construction may lead to different results. Variation in FEV_1_ in these results may be due to differences in population characteristics between the two studies, residual confounding, or as demonstrated by methylation levels, differences in expression levels of detoxification enzymes produced by this set of genes. Based on the results of this study, methylation levels at age 18 within this gene cluster can be ruled out because analyses revealed no significant effect of methylation on lung function levels at 18. The goal of this study was to expand upon findings by Breton *et al.* through accounting for methylation [13].

## Conclusions

Based on results of this study, it is clear that certain genes within the *GSTM2-5* loci explain some differences in lung function in late adolescence; however, joint effects with tobacco smoke exposure were not detected. This finding was validated through examination of joint effects of diplotypes and *in utero* smoke exposure, which did not significantly alter methylation levels within this gene cluster. Only one study has reported an interaction of maternal smoking with *GSTM2-5* genes [13]. A past genetic association study has examined the interactive effects of maternal genotypes on other *GST* genes [12], but no studies have examined the joint effect of maternal genotype and *GSTM2-5* genotypes in their offspring. Future studies are necessary to elucidate conflicting results, taking into account DNA methylation ×gene interactions, interaction with other xenobiotics affecting the glutathione levels, and interaction with the maternal genome.

## Competing interests

The authors declare that they have no competing interests.

## Authors’ contributions

MA performed the research, statistical analyses, data interpretation and wrote the manuscript. WK, JWH, HZ, RJK, GR and SHA performed research, interpreted data, and critically read the manuscript. SE carried out the molecular genetic studies, interpreted data and critically read the manuscript. All authors read and approved the final manuscript.

## Pre-publication history

The pre-publication history for this paper can be accessed here:

http://www.biomedcentral.com/1471-2466/13/56/prepub

## Supplementary Material

Additional file 1 **Sample collection, genotyping methods, and haplotype blocks within *GSTM2-5.*** This additional file contains information on sample collection, genotyping, and haplotype block creation **(Figure S2)** and diplotype frequencies **(Table S1).** Click here for file

Additional file 2 **Statistical analyses and results of SNP effects.** This additional file contains information on statistical methods used to analyze effects of SNPs on lung function growth **(Table S2).** Two plots were created to compare results from the present study with a study by Breton *et al.***(Figures S3** and **S4).** Click here for file

Additional file 3 **Effect of *GSTM2-5 *diplotypes x smoke interactions and CpG sites on lung function levels and lung function growth.** This file contains supplementary tables on main effects of *GSTM2-5* diplotypes on lung function levels at ages 10 and 18 **(Tables S3a** and **S3b)**, interactions between *GSTM2-5* diplotypes and smoke exposures on lung function levels at ages 10 and 18 **(Tables S4a-S4c, Tables S5a-S5c, Tables S6a-S6c,** and **Tables S7a-S7c)**, interactions between *GSTM2-5* diplotypes and smoke exposures on lung function growth **(Tables S8a-S8c, Tables S5a-S5c,** and **Tables S6a-S6c)**, the influence of diplotypes on CpG site methylation **(Table S12)**, the effect of CpG sites on lung function growth **(Table S13)**, validation of smoke exposure categories via urinary cotinine **(Table S15)**, and the effect of active smoking on lung function levels at age 18 **(Table S16).** Click here for file
