# Supplementary material for: Effect of GSTM2-5 polymorphisms in relation to tobacco smoke exposures on lung function growth: a birth cohort study
Source: BMC Pulm Med. 2013 Sep 3;13:56. doi: 10.1186/1471-2466-13-56 (PMC3846453; doi:10.1186/1471-2466-13-56)
Supplement: Additional file 1 — Sample collection, genotyping methods, and haplotype blocks within GSTM2-5. This additional file contains information on sample collection, genotyping, and haplotype block creation (Figure S2) and diplotype frequencies (Table S1). [file 1471-2466-13-56-S1.docx]

**Methods**

**Sample collection and genotyping**

Genomic DNA was isolated from frozen blood samples using QIAamp DNA Blood Kits (Qiagen, Valencia, CA, U.S.A.) or the ABI PRISM™ 6100 Nucleic Acid PrepStation (Applied Biosystems, Foster City, CA, U.S.A.). Polymorphisms were examined using SNPper, HapMap (http://www.hapmap.org/) and Applied Biosystems (https://products.appliedbiosystems.com/) databases. Illumina GoldenGate assays were used to genotype 10 tagging polymorphisms from *GSTM2* (rs574344 and rs12024479), *GSTM3* (rs1537236, rs7483, and rs10735234), *GSTM4* (rs668413, rs560018, and rs506008), and *GSTM5* (rs11807) genes. These SNPs were used to replicate the study conducted by Breton *et al*.[12]

**Path analysis**

To verify whether or not CpG sites that were modified by the joint effect of diplotypes and tobacco smoke exposure acted as an intervening variable between the diplotype and the lung function outcome at age 18, a path analytical model was constructed using the CALIS procedure in SAS. A two-stage estimation was performed. First, unweighted least-squares estimates of the model parameters and their residuals were computed. Estimates were then used as initial values for the optimization process to compute maximum likelihood parameter estimates. The path coefficients represent the partial correlation between the dependent and independent variables adjusted for other covariates. These path coefficients are also known as the direct effects (the effect of a risk factor on an outcome that is not moderated by other variables), indirect effects (the effect of the risk factor on an outcome variable with an intervening variable), and total effects (the sum of direct and indirect effects of the path). Figure S1 illustrates the theoretical model from *GSTM2-5* diplotypes to *GSTM2-5* CpG site methylation to lung function levels at age 18 (Figure S1).

Figure S1Theoretical path analytical model


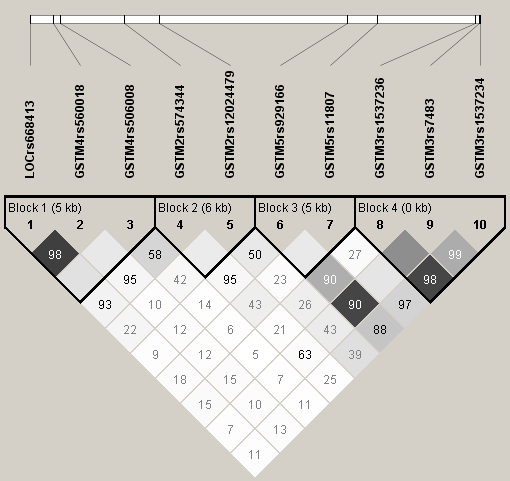


Figure S2 Haploview 3.2 generated linkage disequilibrium (LD) blocks for each of the GSTM2-5 loci with D’ values. Dark grey squares indicate statistically significant (LOD≥2) linkage disequilibrium (LD) between the pair of SNPs, where D’ = 1. Lighter shades of grey also indicate statistically significant LD between a pair of SNPS, where D’ < 1. White squares indicate pairwise D’ values of <1 with no statistically significant evidence of LD.

Table S1 Diplotype frequencies of the Isle of Wight birth cohort

| Gene | Diplotype | Frequency (%) |
| --- | --- | --- |
| GSTM2 | TC_AC | 6.06 |
| (n = 1138) | TC_TC | 20.65 |
|  | TC_TG | 41.48 |
|  | TG_AC | 7.91 |
|  | TG_TG | 23.37 |
|  | Minor diplotypes* | 0.53 |
| GSTM3 | AAA_AAA | 9.26 |
| (n = 1156) | AAA_GGG | 25.52 |
|  | AGA_AAA | 10.55 |
|  | AGA_GGG | 18.25 |
|  | GGA_GGG | 5.8 |
|  | GGG_GGG | 18.51 |
|  | Minor diplotypes | 12.11 |
| GSTM4 | AGG_AGG | 12.37 |
| (n = 1148) | CAA_AGG | 10.19 |
|  | CAG_AAG | 5.23 |
|  | CAG_AGG | 30.75 |
|  | CAG_CAA | 12.11 |
|  | CAG_CAG | 20.21 |
|  | Minor diplotypes | 9.15 |
| GSTM5 | AA_AA | 30.17 |
| (n = 1117) | AA_AG | 21.31 |
|  | AA_CA | 26.23 |
|  | AG_CA | 9.76 |
|  | CA_CA | 8.50 |
|  | Minor diplotypes | 4.02 |

* The following SNPs comprised subsequent blocks: rs668413, rs560018, and rs506008 in GSTM4; rs574344 and rs12024479 in GSTM2; rs929166 and rs11807 in GSTM5; and rs1537236, rs7483, and rs10735234 in GSTM3

†Minor pairs are composed of diplotypes with a frequency <5%
