# Supplementary material for: Effect of GSTM2-5 polymorphisms in relation to tobacco smoke exposures on lung function growth: a birth cohort study
Source: BMC Pulm Med. 2013 Sep 3;13:56. doi: 10.1186/1471-2466-13-56 (PMC3846453; doi:10.1186/1471-2466-13-56)
Supplement: Additional file 2 — Statistical analyses and results of SNP effects. This additional file contains information on statistical methods used to analyze effects of SNPs on lung function growth (Table S2). Two plots were created to compare results from the present study with a study by Breton et al.(Figures S3 and S4). [file 1471-2466-13-56-S2.docx]

**Single SNP statistical analysis**

Single SNPs were assessed using linear mixed models to determine which SNPs were driving the effect of the diplotypes and influencing lung function. Height, BMI, sex, *GSTM1* activity, and age were included as potential confounders. Additive models were created based on the number of minor alleles (0 = no minor alleles; 1 = one minor allele; 2 = two minor alleles). Statistical significance of the SNPs was adjusted for FDR of 0.05 within each lung function outcome. After single SNP analysis, lung function levels in the present study were compared to levels found by Breton *et al* using Spearman’s rho.[12]

**Results**

***GSTM2-5* SNP effects**

Additive models were run to determine the effect of the number of minor alleles on pulmonary function. No SNPs within the *GSTM2-5* cluster demonstrated significant effects on lung function outcomes (Table S2). When these results were compared to the single SNP effects on FEV1 and FVC found in Breton *et al*, there was very little agreement (Figures S2 and S3). FEV_1_ values had a Spearman’s rho (r_s_) of 0.28 (p = 0.43), a weak and insignificant correlation. FVC values had an almost no agreement (r_s_ = -0.08, p = 0.83) (data not shown).

Table S2 Single SNP associations of *GSTM2-5* cluster with lung function outcomes

| Gene | SNP* |  | Forced expiratory volume  in 1 second (FEV_1_) † | | Forced Vital Capacity (FVC) † | | FEV_1_/FVC (%)† | |
| --- | --- | --- | --- | --- | --- | --- | --- | --- |
|  |  | n | β (mL) | p-value | β (mL) | p-value | β (%) | p-value |
| *GSTM2* | rs574344 | 992 | 0.89 | 0.97 | -2.48 | 0.91 | 0.23 | 0.64 |
|  | rs12024479 | 969 | 12.34 | 0.25 | 11.97 | 0.29 | 0.12 | 0.64 |
| *GSTM3* | rs1537236 | 978 | 2.80 | 0.80 | -7.45 | 0.52 | 0.28 | 0.29 |
|  | rs7483 | 982 | 14 | 0.23 | 6.57 | 0.59 | 0.25 | 0.35 |
|  | rs10735234 | 979 | -2.87 | 0.79 | 9.84 | 0.38 | -0.36 | 0.16 |
| *GSTM4* | rs668413 | 965 | 1.33 | 0.90 | -1.12 | 0.92 | 0.18 | 0.47 |
|  | rs560018 | 970 | 0.87 | 0.94 | -2.75 | 0.82 | 0.18 | 0.50 |
|  | rs506008 | 986 | 19.42 | 0.22 | 26.61 | 0.11 | -0.18 | 0.62 |
| *GSTM5* | rs929166 | 946 | 5.54 | 0.65 | 2.63 | 0.83 | 0.03 | 0.92 |
|  | rs11807 | 981 | -14.80 | 0.28 | -18.12 | 0.20 | -0.05 | 0.87 |

* SNPs were analyzed as a continuous variable, where zero, one, or two represent the number of variant alleles;

†Models adjusted for sex, body mass index (BMI), height, current smoking, and current asthma status

**
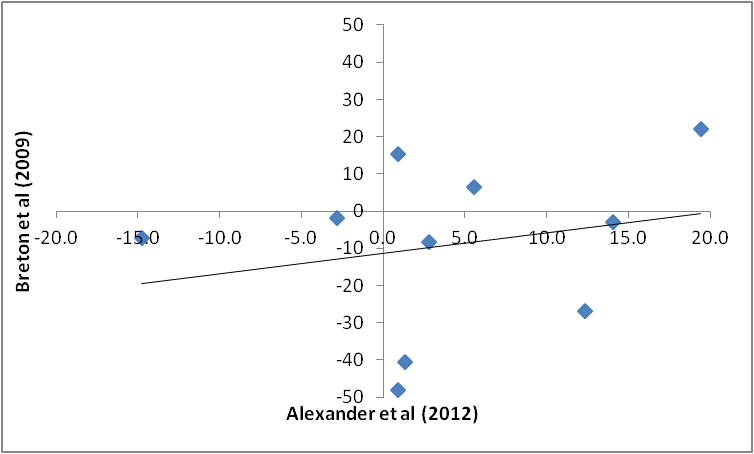
**

Figure S3 Scatterplot of associations between single nucleotide polymorphisms (SNPs) in the GSTM2-5 loci and growth in FEV_1_ (mL) in the present study (horizontal axis) and Breton et al (vertical axis) studies. Models in the present study were adjusted for sex, body mass index (BMI), height, current smoking, and current asthma status.

**
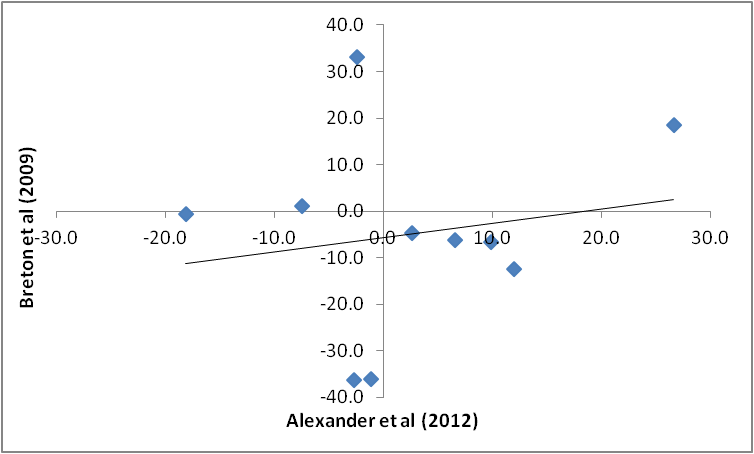
**

Figure S4 Scatterplot of associations between single nucleotide polymorphisms (SNPs) in the GSTM2-5 loci and growth in FVC (mL) in Alexander et al (horizontal axis) and Breton et al (vertical axis) studies. Models in the present study were adjusted for sex, body mass index (BMI), height, current smoking, and current asthma status.
