# Supplementary material for: Effect of GSTM2-5 polymorphisms in relation to tobacco smoke exposures on lung function growth: a birth cohort study
Source: BMC Pulm Med. 2013 Sep 3;13:56. doi: 10.1186/1471-2466-13-56 (PMC3846453; doi:10.1186/1471-2466-13-56)
Supplement: Additional file 3 — Effect of GSTM2-5 diplotypes x smoke interactions and CpG sites on lung function levels and lung function growth. This file contains supplementary tables on main effects of GSTM2-5 diplotypes on lung function levels at ages 10 and 18 (Tables S3a and S3b), interactions between GSTM2-5 diplotypes and smoke exposures on lung function levels at ages 10 and 18 (Tables S4a-S4c, Tables S5a-S5c, Tables S6a-S6c, and Tables S7a-S7c), interactions between GSTM2-5 diplotypes and smoke exposures on lung function growth (Tables S8a-S8c, Tables S5a-S5c, and Tables S6a-S6c), the influence of diplotypes on CpG site methylation (Table S12), the effect of CpG sites on lung function growth (Table S13), validation of smoke exposure categories via urinary cotinine (Table S15), and the effect of active smoking on lung function levels at age 18 (Table S16). [file 1471-2466-13-56-S3.docx]

Table S3a Adjusted effects of diplotypes within *GSTM2-5* on lung function outcomes at age 10 years

|  |  |  | Forced expiratory volume in 1 second (FEV_1_) | | Forced vital capacity (FVC) | | FEV_1_/FVC | |
| --- | --- | --- | --- | --- | --- | --- | --- | --- |
| Gene | n | Diplotype* | β (mL) | p-value§ | β (mL) | p-value§ | β (%) | p-value§ |
| *GSTM2* | 868 | TC_AC | -4.78 | 0.97 | -25.72 | 0.75 | 0.83 | 0.56 |
|  |  | TC_TC | -31.28 | 0.60 | -45.07 | 0.15 | 0.44 | 0.56 |
|  |  | TG_AC | 1.01 | 0.97 | -10.13 | 0.75 | 0.46 | 0.56 |
|  |  | TG_TG | 2.34 | 0.97 | -11.18 | 0.75 | 0.58 | 0.56 |
|  |  | Minor diplotypes | 113.03 | 0.95 | 59.47 | 0.75 | 2.39 | 0.56 |
|  |  | TC_TG | REF | -- | REF | -- | REF | -- |
|  |  | p-value‡ | 0.58 | | 0.39 | | 0.79 | |
| *GSTM3* | 886 | AAA_AAA | -43.52 | 0.18 | -44.96 | 0.16 | 0.01 | 0.99 |
|  |  | AGA_AAA | -43.88 | 0.18 | **-70.92** | 0.06 | 0.88 | 0.63 |
|  |  | AGA_GGG | -32.58 | 0.19 | -30.36 | 0.21 | -0.17 | 0.94 |
|  |  | GGA_GGG | -37.44 | 0.28 | -60.59 | 0.14 | 0.53 | 0.94 |
|  |  | GGG_GGG | **-65.04** | **0.03** | **-50.61** | 0.09 | -0.84 | 0.63 |
|  |  | Minor diplotypes | -43.17 | 0.18 | -46.25 | 0.14 | -0.21 | 0.94 |
|  |  | AAA_GGG | REF | -- | REF | -- | REF | -- |
|  |  | p-value‡ | 0.18 | | 0.17 | | 0.40 | |
| *GSTM4* | 877 | AGG_AGG | 10.52 | 0.87 | 39.12 | 0.34 | -0.94 | 0.26 |
|  |  | CAA_AGG | 17.22 | 0.87 | 31.42 | 0.39 | -0.34 | 0.74 |
|  |  | CAG_AAG | -5.77 | 0.87 | -10.15 | 0.79 | 0.22 | 0.82 |
|  |  | CAG_CAA | 14.35 | 0.87 | 39.64 | 0.34 | -0.89 | 0.26 |
|  |  | CAG_CAG | -4.07 | 0.87 | 24.72 | 0.40 | -0.92 | 0.26 |
|  |  | Minor diplotypes | 7.89 | 0.87 | 41.13 | 0.34 | -1.23 | 0.26 |
|  |  | CAG_AGG | REF | -- | REF | -- | REF | -- |
|  |  | p-value‡ | 0.98 | | 0.52 | | 0.42 | |
| *GSTM5* | 852 | AA_AG | 28.83 | 0.45 | 9.61 | 0.76 | 0.97 | 0.20 |
|  |  | AA_CA | **59.98** | **0.02** | 38.42 | 0.35 | 1.08 | 0.20 |
|  |  | AG_CA | -13.50 | 0.79 | -22.58 | 0.73 | 0.30 | 0.97 |
|  |  | CA_CA | 2.22 | 0.94 | 9.38 | 0.76 | -0.11 | 0.97 |
|  |  | Minor diplotypes | -30.32 | 0.75 | -42.74 | 0.73 | 0.04 | 0.97 |
|  |  | AA_AA | REF | -- | REF | -- | REF | -- |
|  |  | p-value‡ | **0.02** | | 0.22 | | 0.23 | |

* Comprised of SNPs as described in the Results section; †Models adjusted for sex, rs366631, *in utero* smoke exposure, current secondhand smoke exposure, body mass index (BMI) and height; ‡ Global F-test p-value: combined effect of diplotypes on repeated lung function measurements; § After adjustment for false discovery rate (FDR)

Table S3b Adjusted effects of diplotypes within *GSTM2-5* on lung function outcomes at age 18 years

|  |  |  | Forced expiratory volume in 1 second (FEV_1_) | | Forced Vital Capacity (FVC) | | FEV_1_/FVC † | |
| --- | --- | --- | --- | --- | --- | --- | --- | --- |
| Gene | n | Diplotype* | β (mL) | p-value§ | β (mL) | p-value§ | β (%) | p-value§ |
| *GSTM2* | 731 | TC_AC | 67.78 | 0.70 | 26.94 | 0.96 | 0.67 | 0.74 |
|  |  | TC_TC | 33.64 | 0.70 | 84.57 | 0.50 | -0.65 | 0.74 |
|  |  | TG_AC | 63.06 | 0.70 | 22.37 | 0.96 | 0.86 | 0.74 |
|  |  | TG_TG | -4.59 | 0.92 | 0.49 | 0.99 | -0.02 | 0.98 |
|  |  | Minor diplotypes | 137.74 | 0.70 | 76.99 | 0.96 | 2.13 | 0.74 |
|  |  | TC_TG | REF | -- | REF | -- | REF | -- |
|  |  | p-value‡ | 0.83 | | 0.68 | | 0.79 | |
| *GSTM3* | 742 | AAA_AAA | 22.16 | 0.89 | -73.45 | 0.68 | 2.03 | 0.18 |
|  |  | AGA_AAA | -28.98 | 0.89 | -63.88 | 0.68 | 0.63 | 0.60 |
|  |  | AGA_GGG | 69.31 | 0.89 | 25.46 | 0.68 | 1.27 | 0.18 |
|  |  | GGA_GGG | 69.33 | 0.89 | -38.00 | 0.68 | 2.25 | 0.18 |
|  |  | GGG_GGG | -3.85 | 0.94 | -56.14 | 0.68 | 1.27 | 0.18 |
|  |  | Minor diplotypes | 39.88 | 0.89 | 34.16 | 0.68 | 0.23 | 0.82 |
|  |  | AAA_GGG | REF | -- | REF | -- | REF | -- |
|  |  | p-value‡ | 0.72 | | 0.63 | | 0.26 | |
| *GSTM4* | 739 | AGG_AGG | -14.86 | 0.91 | -44.33 | 0.94 | 0.56 | 0.83 |
|  |  | CAA_AGG | 138.92 | 0.12 | 139.64 | 0.24 | 0.37 | 0.83 |
|  |  | CAG_AAG | 16.45 | 0.91 | 6.59 | 0.94 | 0.29 | 0.83 |
|  |  | CAG_CAA | 57.94 | 0.91 | 14.32 | 0.94 | 0.71 | 0.83 |
|  |  | CAG_CAG | 5.81 | 0.91 | 18.29 | 0.94 | -0.27 | 0.83 |
|  |  | Minor diplotypes | 27.69 | 0.91 | 58.16 | 0.94 | -0.73 | 0.83 |
|  |  | CAG_AGG | REF | -- | REF | -- | REF | -- |
|  |  | p-value‡ | 0.35 | | 0.38 | | 0.87 | |
| *GSTM5* | 716 | AA_AG | 61.63 | 0.53 | 97.45 | 0.35 | -0.58 | 0.61 |
|  |  | AA_CA | -5.22 | 0.91 | 0.95 | 0.99 | -0.49 | 0.61 |
|  |  | AG_CA | -107.11 | 0.50 | 10.25 | 0.99 | -2.25 | 0.15 |
|  |  | CA_CA | 22.08 | 0.91 | 69.46 | 0.88 | -0.84 | 0.61 |
|  |  | Minor diplotypes | -61.66 | 0.91 | -74.84 | 0.88 | -0.14 | 0.93 |
|  |  | AA_AA | REF | -- | REF | -- | REF | -- |
|  |  | p-value‡ | 0.24 | | 0.38 | | 0.39 | |

* Comprised of SNPs as described in the Results section; †Models adjusted for sex, rs366631, *in utero* smoke exposure, current secondhand smoke exposure, active smoking, body mass index (BMI) and height; ‡ Global F-test p-value: combined effect of diplotypes on repeated lung function measurements; § After adjustment for false discovery rate (FDR)

Table S4a Adjusted estimates of diplotypes within *GSTM2* on lung function measurements at ages 10 years (n = 868) and 18 years (n = 731) by *in utero* smoke exposure status

|  | Forced expiratory volume in 1 second (FEV_1_) (mL) at age 10† | | Forced Vital Capacity  (FVC) (mL) at age 10 † | | FEV_1_/FVC (%) at age 10† | |
| --- | --- | --- | --- | --- | --- | --- |
| Diplotype* | Not exposed  (β, SE) | Exposed  (β, SE) | Not exposed  (β, SE) | Exposed  (β, SE) | Not exposed  (β, SE) | Exposed  (β, SE) |
| TC_AC | -4.22 (37.99) | -4.40 (59.00) | 6.39 (40.48) | -113.30 (56.79) | -0.31 (0.96) | 4.03 (1.73) |
| TC_TC | -44.81 (23.00) | 20.04 (40.39) | -52.63 (24.44) | -12.01 (38.89) | 0.18 (0.58) | 1.27 (1.18) |
| TG_AC | 7.36 (35.27) | -21.66 (60.36) | -3.41 (37.58) | -38.59 (58.10) | 0.41 (0.89) | 0.78 (1.77) |
| TG_TG | -12.73 (22.43) | 58.67 (39.86) | -18.48 (23.90) | 16.27 (38.37) | 0.23 (0.57) | 1.89 (1.17) |
| Minor diplotypes | 106.26 (130.35) | -- | 58.13 (138.89) | -- | 2.14 (3.29) | -- |
| TC_TG | REF | REF | REF | REF | REF | REF |
| p_interaction_‡ | 0.46 | | 0.53 | | 0.28 | |
|  | Forced expiratory volume in 1 second (FEV_1_) (mL) at age 18† | | Forced Vital Capacity  (FVC) (mL) at age 18 † | | FEV_1_/FVC (%) at age 18† | |
| Diplotype* | Not exposed  (β, SE) | Exposed  (β, SE) | Not exposed  (β, SE) | Exposed  (β, SE) | Not exposed  (β, SE) | Exposed  (β, SE) |
| TC_AC | 140.52 (91.89) | -131.02 (173.95) | 144.80 (99.94) | -366.53 (191.28) | 0.08 (1.39) | 2.59 (2.86) |
| TC_TC | 14.82 (52.73) | 119.67 (102.00) | 61.85 (57.37) | 193.40 (111.00) | -0.71 (0.80) | -0.52 (1.66) |
| TG_AC | 26.24 (80.28) | 207.05 (144.74) | 24.55 (87.49) | 22.12 (162.37) | -0.12 (1.22) | 3.76 (2.36) |
| TG_TG | -20.96 (49.15) | 80.72 (103.26) | 3.73 (53.46) | 2.18 (112.51) | -0.39 (0.74) | 1.48 (1.68) |
| Minor diplotypes | 122.37 (237.26) | -- | 79.74 (258.64) | -- | 1.62 (3.60) | -- |
| TC_TG | REF | REF | REF | REF | REF | REF |
| p_interaction_‡ | 0.33 | | 0.10 | | 0.43 | |

* Comprised of SNPs as described in the Results section; †Models adjusted for sex, rs366631, current secondhand smoke exposure, current body mass index (BMI), and current height; additional adjustment for current smoking for lung function levels at age 18; -- not estimable due to small size; ‡ Global F-test p-value: significance of interaction

Table S4b Adjusted estimates of diplotypes within *GSTM2* on lung function measurements at ages 10 years (n = 868) and 18 years (n = 731) by secondhand smoke exposure status

|  | Forced expiratory volume in 1 second (FEV_1_) (mL) at age 10† | | Forced Vital Capacity  (FVC) (mL) at age 10 † | | FEV_1_/FVC (%) at age 10† | |
| --- | --- | --- | --- | --- | --- | --- |
| Diplotype* | Not exposed  (β, SE) | Exposed  (β, SE) | Not exposed  (β, SE) | Exposed  (β, SE) | Not exposed  (β, SE) | Exposed  (β, SE) |
| TC_AC | 17.07 (49.68) | -25.54 (42.00) | 26.09 (52.47) | -66.62 (43.49) | 0.14 (1.28) | 1.27 (1.11) |
| TC_TC | -49.82 (29.08) | -12.02 (27.60) | 59.13 (30.71) | -31.35 (28.47) | 0.30 (0.75) | 0.64 (0.73) |
| TG_AC | 0.72 (44.16) | 3.64 (42.46) | -18.76 (46.64) | 1.49 (43.96) | 0.89 (1.14) | 0.04 (1.12) |
| TG_TG | -7.24 (27.82) | 9.18 (27.63) | -12.80 (29.38) | -14.86 (28.61) | 0.33 (0.72) | 0.93 (0.73) |
| Minor diplotypes | 374.14 (221.51) | -28.29 (157.18) | 200.63 (233.94) | -15.52 (162.74) | 8.22 (5.70) | -0.84 (4.16) |
| TC_TG | REF | REF | REF | REF | REF | REF |
| p_interaction_‡ | 0.65 | | 0.81 | | 0.85 | |
|  | Forced expiratory volume in 1 second (FEV_1_) (mL) at age 18† | | Forced Vital Capacity  (FVC) (mL) at age 18 † | | FEV_1_/FVC (%) at age 18† | |
| Diplotype* | Not exposed  (β, SE) | Exposed  (β, SE) | Not exposed  (β, SE) | Exposed  (β, SE) | Not exposed  (β, SE) | Exposed  (β, SE) |
| TC_AC | 98.14 (118.01) | 32.85 (111.19) | 236.63 (122.63) | -167.82 (124.60) | -2.07 (1.84) | 2.94 (1.71) |
| TC_TC | 4.04 (72.45) | 56.27 (60.62) | 106.05 (75.29) | 71.77 (67.94) | -1.52 (1.14) | -0.07 (0.93) |
| TG_AC | 25.72 (101.06) | 103.86 (98.10) | 4.48 (105.02) | 36.99 (111.29) | 0.34 (1.58) | 1.54 (1.51) |
| TG_TG | -25.15 (63.47) | 11.79 (60.75) | -18.66 (65.95) | 14.18 (68.07) | -0.13 (1.00) | 0.01 (0.93) |
| Minor diplotypes | -1429.75 (462.66) | 684.23 (275.31) | -1737.93 (480.78) | 668.66 (308.53) | 2.48 (7.23) | 2.56 (4.24) |
| TC_TG | REF | REF | REF | REF | REF | REF |
| p_interaction_‡ | **0.004** | | **0.0003** | | 0.84 | |

* Comprised of SNPs as described in the Results section; †Models adjusted for sex, rs366631, *in utero* exposure, current body mass index (BMI), current height; additional adjustment for current smoking for lung function levels at age 18; ‡ Global F-test p-value: significance of interaction

Table S4c Adjusted estimates of diplotypes within *GSTM2* on lung function measurements at age 18 years by personal smoking status (n = 731)

|  | Forced expiratory volume in 1 second (FEV_1_) (mL) at age 18† | | Forced Vital Capacity  (FVC) (mL) at age 18 † | | FEV_1_/FVC (%) at age 18† | |
| --- | --- | --- | --- | --- | --- | --- |
| Diplotype* | Not exposed  (β, SE) | Exposed  (β, SE) | Not exposed  (β, SE) | Exposed  (β, SE) | Not exposed  (β, SE) | Exposed  (β, SE) |
| TC_AC | -6.78 (90.62) | 337.11 (181.45) | 43.17 (98.78) | -18.95 (198.03) | -1.14 (1.41) | 6.80 (2.63) |
| TC_TC | 17.19 (54.77) | 72.90 (91.15) | 75.30 (59.71) | 86.03 (99.48) | -0.98 (0.85) | 0.47 (1.32) |
| TG_AC | 75.13 (76.22) | -24.27 (179.80) | 50.41 (83.14) | -88.17 (205.22) | 0.40 (1.19) | 1.69 (2.60) |
| TG_TG | -27.08 (50.47) | 47.91 (93.20) | -8.73 (55.01) | -16.47 (101.71) | -0.57 (0.79) | 2.07 (1.35) |
| Minor diplotypes | 124.37 (233.31) | -- | 70.38 (254.31) | -- | 1.62 (3.64) | -- |
| TC_TG | REF | REF | REF | REF | REF | REF |
| p_interaction_‡ | 0.25 | | 0.89 | | 0.29 | |

* Comprised of SNPs as described in the Results section; †Models adjusted for sex, rs366631, *in utero* smoke exposure, current secondhand smoke exposure, current body mass index (BMI), and current height; ‡ Global F-test p-value: significance of interaction

Table S5a Adjusted estimates of diplotypes within *GSTM3* on lung function measurements at age 10 years (n = 886) and 18 years (n = 742) by *in utero* smoke exposure status

|  | Forced expiratory volume in 1 second (FEV_1_) (mL) at age 10† | | Forced Vital Capacity  (FVC) (mL) at age 10 † | | FEV_1_/FVC (%) at age 10† | |
| --- | --- | --- | --- | --- | --- | --- |
| Diplotype* | Not exposed  (β, SE) | Exposed  (β, SE) | Not exposed  (β, SE) | Exposed  (β, SE) | Not exposed  (β, SE) | Exposed  (β, SE) |
| AAA_AAA | -35.15 (33.28) | -65.90 (53.71) | -38.53 (35.46) | -62.22 (52.04) | 0.05 (0.84) | -0.09 (1.58) |
| AGA_AAA | -62.56 (30.58) | 36.55 (59.83) | -84.49 (32.59) | -15.09 (57.97) | 0.67 (0.77) | 1.86 (1.76) |
| AGA_GGG | -41.67 (26.09) | 8.46 (48.59) | -40.76 (27.81) | 15.12 (47.08) | -0.18 (0.66) | -0.07 (1.43) |
| GGA_GGG | -29.60 (41.18) | -34.10 (64.61) | -57.15 (43.84) | -44.77 (62.61) | 0.65 (1.04) | 0.33 (1.90) |
| GGG_GGG | -70.87 (25.96) | -37.22 (48.67) | -62.40 (27.67) | 0.80 (47.16) | -0.70 (0.66) | -1.35 (1.43) |
| Minor diplotypes | -64.00 (30.53) | 30.82 (53.35) | -64.03 (32.39) | 19.15 (51.69) | -0.45 (0.78) | 0.62 (0.16) |
| AAA_GGG | REF | REF | REF | REF | REF | REF |
| p_interaction_‡ | 0.54 | | 0.90 | | 0.96 | |
|  | Forced expiratory volume in 1 second (FEV_1_) (mL) at age 18† | | Forced Vital Capacity  (FVC) (mL) at age 18 † | | FEV_1_/FVC (%) at age 18† | |
| Diplotype* | Not exposed  (β, SE) | Exposed  (β, SE) | Not exposed  (β, SE) | Exposed  (β, SE) | Not exposed  (β, SE) | Exposed  (β, SE) |
| AAA_AAA | 16.21 (76.35) | 37.66 (130.56) | -96.52 (83.25) | 35.11 (148.19) | 2.15 (1.16) | 11.82 (21.40) |
| AGA_AAA | -51.61 (69.00) | 111.47 (137.15) | -102.45 (75.23) | 144.29 (155.71) | 1.01 (1.05) | -5.07 (22.48) |
| AGA_GGG | 80.55 (58.90) | 73.71 (117.80) | 13.94 (64.22) | 118.51 (134.13) | 1.67 (0.89) | 0.47 (19.30) |
| GGA_GGG | 83.87 (94.80) | 54.63 (180.71) | -45.46 (103.37) | 60.39 (205.31) | 2.77 (1.44) | 2.79 (29.61) |
| GGG_GGG | -6.05 (57.40) | -11.99 (111.00) | -93.39 (62.59) | 94.97 (126.38) | 1.84 (0.87) | -12.21 (18.19) |
| Minor diplotypes | 17.57 (71.67) | 113.92 (147.67) | -9.88 (78.14) | 233.64 (168.43) | 0.57 (1.09) | -17.16 (24.20) |
| AAA_GGG | REF | REF | REF | REF | REF | REF |
| p_interaction_‡ | 0.94 | | 0.89 | | 0.96 | |

* Comprised of SNPs as described in the Results section; †Models adjusted for sex, rs366631, current smoking, current secondhand smoke exposure, current body mass index (BMI), and current height; additional adjustment for current smoking for lung function levels at age 18; ‡ Global F-test p-value: significance of interaction

Table S5b Adjusted estimates of diplotypes within *GSTM3* on lung function measurements at ages 10 years (n = 886) and 18 years (n = 742) by secondhand smoke exposure status

|  | Forced expiratory volume in 1 second (FEV_1_) (mL) at age 10† | | Forced Vital Capacity  (FVC) (mL) at age 10 † | | FEV_1_/FVC (%) at age 10† | |
| --- | --- | --- | --- | --- | --- | --- |
| Diplotype* | Not exposed  (β, SE) | Exposed  (β, SE) | Not exposed  (β, SE) | Exposed  (β, SE) | Not exposed  (β, SE) | Exposed  (β, SE) |
| AAA_AAA | -65.83 (41.32) | -21.13 (38.98) | -60.57 (43.47) | -24.58 (40.52) | -0.42 (1.07) | 0.25 (1.02) |
| AGA_AAA | -63.84 (40.51) | -33.51 (36.97) | -85.53 (42.61) | -63.43 (38.42) | 0.57 (1.05) | 1.04 (0.97) |
| AGA_GGG | -34.20 (31.86) | -34.18 (33.28) | -45.76 (33.51) | -13.61 (34.60) | 0.30 (0.82) | -0.80 (0.87) |
| GGA_GGG | -30.85 (51.02) | -40.35 (47.39) | -49.73 (53.66) | -61.98 (49.25) | 0.36 (1.32) | 0.51 (1.24) |
| GGG_GGG | -78.55 (32.91) | -52.35 (31.89) | -80.14 (34.62) | -21.25 (33.14) | -0.25 (0.85) | -1.46 (0.84) |
| Minor diplotypes | -90.33 (40.76) | -8.87 (35.19) | -83.90 (42.87) | -14.56 (36.39) | -0.92 (1.05) | 0.14 (0.92) |
| AAA_GGG | REF | REF | REF | REF | REF | REF |
| p_interaction_‡ | 0.70 | | 0.92 | | 0.55 | |
|  | Forced expiratory volume in 1 second (FEV_1_) (mL) at age 18† | | Forced Vital Capacity  (FVC) (mL) at age 18 † | | FEV_1_/FVC (%) at age 18† | |
| Diplotype* | Not exposed  (β, SE) | Exposed  (β, SE) | Not exposed  (β, SE) | Exposed  (β, SE) | Not exposed  (β, SE) | Exposed  (β, SE) |
| AAA_AAA | -98.79 (104.12) | 98.69 (85.90) | -170.50 (110.56) | -1.55 (97.33) | 0.85 (1.61) | 2.60 (1.31) |
| AGA_AAA | 5.10 (93.61) | -52.72 (81.58) | -79.32 (99.40) | -47.35 (92.42) | 1.74 (1.45) | -0.20 (1.25) |
| AGA_GGG | 103.78 (77.72) | 36.43 (71.44) | -33.37 (82.53) | 65.93 (81.00) | 2.94 (1.20) | -0.09 (1.09) |
| GGA_GGG | 235.99 (113.39) | -149.06 (124.06) | 24.74 (120.40) | -146.50 (140.59) | 4.95 (1.76) | -0.85 (1.90) |
| GGG_GGG | -39.58 (75.05) | 24.04 (69.61) | -132.34 (79.69) | 6.32 (78.95) | 2.19 (1.16) | 0.46 (1.07) |
| Minor diplotypes | -36.80 (99.50) | 84.63 (84.86) | -89.93 (105.65) | 116.14 (96.27) | 0.68 (1.54) | -0.23 (1.30) |
| AAA_GGG | REF | REF | REF | REF | REF | REF |
| p_interaction_‡ | **0.04** | | 0.60 | | 0.16 | |

* Comprised of SNPs as described in the Results section; †Models adjusted for sex, rs366631, *in utero* exposure, current body mass index (BMI), current height; additional adjustment for current smoking for lung function levels at age 18 ‡ Global F-test p-value: significance of interaction

Table S5c Adjusted estimates of diplotypes within *GSTM3* on lung function measurements at age 18 years by personal smoking status (n = 742)

|  | Forced expiratory volume in 1 second (FEV_1_) (mL) at age 18† | | Forced Vital Capacity  (FVC) (mL) at age 18 † | | FEV_1_/FVC (%) at age 18† | |
| --- | --- | --- | --- | --- | --- | --- |
| Diplotype* | Not exposed  (β, SE) | Exposed  (β, SE) | Not exposed  (β, SE) | Exposed  (β, SE) | Not exposed  (β, SE) | Exposed  (β, SE) |
| AAA_AAA | 66.54 (70.75) | -54.12 (127.25) | -66.15 (84.79) | -24.13 (140.71) | 2.67 (1.20) | 0.13 (1.94) |
| AGA_AAA | -56.15 (77.83) | 71.15 (125.18) | -96.05 (77.08) | 83.79 (138.42) | 065 (1.10) | 0.18 (1.91) |
| AGA_GGG | 68.41 (59.19) | 80.43 (115.14) | -4.18 (64.48) | 132.84 (127.71) | 1.60 (0.92) | 0.25 (1.75) |
| GGA_GGG | 75.72 (93.73) | 47.88 (188.65) | -83.25 (102.11) | 103.49 (209.59) | 3.29 (1.45) | -1.13 (2.87) |
| GGG_GGG | -51.06 (58.00) | 135.02 (107.13) | -127.74 (63.19) | 165.46 (118.77) | 1.57 (0.90) | 0.25 (1.63) |
| Minor diplotypes | -28.65 (73.82) | 235.96 (133.19) | -61.80 (80.42) | 322.84 (147.97) | 0.56 (1.14) | -0.94 (2.03) |
| AAA_GGG | REF | REF | REF | REF | REF | REF |
| p_interaction_‡ | 0.14 | | 0.21 | | 0.81 | |

* Comprised of SNPs as described in the Results section; †Models adjusted for sex, rs366631, *in utero* smoke exposure, current secondhand smoke exposure, current body mass index (BMI), and current height; ‡ Global F-test p-value: significance of interaction

Table S6a Adjusted estimates of diplotypes within *GSTM4* on lung function measurements at ages 10 years (n = 877) and 18 years (n = 739) by *in utero* smoke exposure status

|  | Forced expiratory volume in 1 second (FEV_1_) (mL) at age 10† | | Forced Vital Capacity  (FVC) (mL) at age 10 † | | FEV_1_/FVC (%) at age 10† | |
| --- | --- | --- | --- | --- | --- | --- |
| Diplotype* | Not exposed  (β, SE) | Exposed  (β, SE) | Not exposed  (β, SE) | Exposed  (β, SE) | Not exposed  (β, SE) | Exposed  (β, SE) |
| AGG_AGG | 9.27 (28.72) | 18.47 (53.30) | 35.63 (30.47) | 53.81 (51.81) | -0.92 (0.72) | -0.93 (1.56) |
| CAA_AGG | 32.20 (30.44) | -29.25 (53.04) | 47.97 (32.29) | -20.69 (51.55) | -0.30 (0.77) | -0.44 (1.55) |
| CAG_AAG | 3.83 (40.39) | -44.27 (83.38) | 6.56 (42.85) | -84.82 (81.04) | 0.04 (1.02) | 1.16 (2.44) |
| CAG_CAA | 14.67 (29.04) | 13.19 (48.77) | 53.57 (30.81) | -4.76 (47.40) | -1.42 (0.73) | 0.81 (1.43) |
| CAG_CAG | 0.54 (25.12) | -12.09 (41.78) | 14.88 (26.58) | 46.02 (40.61) | -0.53 (0.63) | -1.99 (1.22) |
| Minor diplotypes | 2.04 (32.51) | 25.99 (61.99) | 36.56 (34.49) | 54.04 (60.25) | -1.26 (0.82) | -1.18 (1.82) |
| CAG_AGG | REF | REF | REF | REF | REF | REF |
| p_interaction_‡ | 0.93 | | 0.79 | | 0.33 | |
|  | Forced expiratory volume in 1 second (FEV_1_) (mL) at age 18† | | Forced Vital Capacity  (FVC) (mL) at age 18 † | | FEV_1_/FVC (%) at age 18† | |
| Diplotype* | Not exposed  (β, SE) | Exposed  (β, SE) | Not exposed  (β, SE) | Exposed  (β, SE) | Not exposed  (β, SE) | Exposed  (β, SE) |
| AGG_AGG | -37.31 (63.53) | 77.42 (145.66) | -72.03 (68.85) | 109.12 (165.14) | 0.76 (0.98) | -0.89 (2.39) |
| CAA_AGG | 171.64 (68.84) | 19.44 (135.03) | 192.79 (74.61) | -49.60 (153.10) | 0.08 (1.06) | 1.36 (2.22) |
| CAG_AAG | 9.97 (91.51) | 55.29 (221.66) | 27.62 (99.18) | -103.90 (251.30) | -0.05 (1.41) | 2.53 (3.64) |
| CAG_CAA | 42.31 (63.51) | 95.84 (117.97) | 21.66 (68.84) | -6.48 (135.65) | 0.33 (0.98) | 1.49 (1.94) |
| CAG_CAG | 36.86 (55.62) | -71.09 (103.42) | 54.35 (60.29) | -78.94 (117.25) | -0.05 (0.86) | -0.80 (1.70) |
| Minor diplotypes | -9.01 (73.49) | 132.71 (158.99) | 45.65 (79.66) | 102.48 (180.29) | -1.11 (1.13) | -0.10 (2.61) |
| CAG_AGG | REF | REF | REF | REF | REF | REF |
| p_interaction_‡ | 0.72 | | 0.77 | | 0.89 | |

* Comprised of SNPs as described in the Results section; †Models adjusted for sex, rs366631, current smoking, current secondhand smoke exposure, current body mass index (BMI), and current height; additional adjustment for current smoking for lung function levels at age 18; ‡ Global F-test p-value: significance of interaction

Table S6b Adjusted estimates of diplotypes within *GSTM4* on lung function measurements at ages 10 years (n = 877) and 18 years (n = 739) by secondhand smoke exposure status

|  | Forced expiratory volume in 1 second (FEV_1_) (mL) at age 10† | | Forced Vital Capacity  (FVC) (mL) at age 10 † | | FEV_1_/FVC (%) at age 10† | |
| --- | --- | --- | --- | --- | --- | --- |
| Diplotype* | Not exposed  (β, SE) | Exposed  (β, SE) | Not exposed  (β, SE) | Exposed  (β, SE) | Not exposed  (β, SE) | Exposed  (β, SE) |
| AGG_AGG | 7.11 (36.32) | 18.56 (35.46) | 22.53 (37.90) | 64.29 (37.01) | -0.66 (0.93) | -1.30 (0.94) |
| CAA_AGG | 40.94 (38.92) | 3.51 (36.21) | 80.86 (40.62) | -6.12 (37.79) | -1.08 (1.00) | 0.39 (0.96) |
| CAG_AAG | 13.71 (54.69) | -18.14 (48.45) | 16.75 (57.07) | -30.48 (50.57) | -0.05 (1.40) | 0.52 (1.28) |
| CAG_CAA | -15.08 (36.95) | 40.99 (34.08) | 31.75 (38.57) | 46.06 (35.57) | -1.71 (0.95) | -0.10 (0.90) |
| CAG_CAG | -15.75 (31.82) | 7.05 (29.36) | 16.32 (33.20) | 30.80 (30.53) | -1.35 (0.81) | -0.66 (0.78) |
| Minor diplotypes | -6.58 (40.62) | 15.80 (40.92) | 17.57 (42.39) | 57.01 (42.71) | -0.88 (1.04) | -1.59 (1.08) |
| CAG_AGG | REF | REF | REF | REF | REF | REF |
| p_interaction_‡ | 0.80 | | 0.62 | | 0.49 | |
|  | Forced expiratory volume in 1 second (FEV_1_) (mL) at age 18† | | Forced Vital Capacity  (FVC) (mL) at age 18 † | | FEV_1_/FVC (%) at age 18† | |
| Diplotype* | Not exposed  (β, SE) | Exposed  (β, SE) | Not exposed  (β, SE) | Exposed  (β, SE) | Not exposed  (β, SE) | Exposed  (β, SE) |
| AGG_AGG | -33.06 (84.91) | -4.93 (78.95) | -173.11 (88.15) | 66.90 (89.11) | 2.63 (1.33) | -1.33 (1.22) |
| CAA_AGG | 221.64 (91.23) | 72.76 (83.31) | 249.61 (94.71) | 46.34 (94.02) | -0.04 (1.44) | 0.86 (1.28) |
| CAG_AAG | 0.74 (110.40) | 13.81 (131.37) | -10.13 (114.61) | 7.04 (148.25) | 0.43 (1.74) | -0.05 (2.02) |
| CAG_CAA | 6.24 (85.35) | 104.04 (74.49) | -49.28 (88.60) | 58.96 (84.51) | 0.73 (1.35) | 0.95 (1.15) |
| CAG_CAG | -14.97 (72.98) | 25.74 (66.51) | 42.93 (75.77) | -6.84 (75.07) | -1.08 (1.15) | 0.61 (1.02) |
| Minor diplotypes | -180.16 (101.08) | 178.22 (88.80) | -83.84 (104.94) | 151.30 (100.23) | -2.52 (1.59) | 0.74 (1.37) |
|  | REF | REF | REF | REF | REF | REF |
| p_interaction_‡ | 0.48 | | 0.68 | | 0.17 | |

* Comprised of SNPs as described in the Results section; †Models adjusted for sex, rs366631, *in utero* exposure, current body mass index (BMI), current height; additional adjustment for current smoking for lung function levels at age 18 ‡ Global F-test p-value: significance of interaction

Table S6c Adjusted estimates of diplotypes within *GSTM4* on lung function measurements at age 18 years by personal smoking status (n = 739)

|  | Forced expiratory volume in 1 second (FEV_1_) (mL) at age 18† | | Forced Vital Capacity  (FVC) (mL) at age 18 † | | FEV_1_/FVC (%) at age 18† | |
| --- | --- | --- | --- | --- | --- | --- |
| Diplotype* | Not exposed  (β, SE) | Exposed  (β, SE) | Not exposed  (β, SE) | Exposed  (β, SE) | Not exposed  (β, SE) | Exposed  (β, SE) |
| AGG_AGG | -5.20 (65.74) | -94.97 (123.29) | -62.37 (71.06) | -54.05 (136.72) | 1.04 (1.04) | +0.57 (1.88) |
| CAA_AGG | 161.65 (71.65) | -5.39 (121.31) | 209.37 (77.44) | -37.89 (134.48) | 0.16 (1.13) | 0.87 (1.85) |
| CAG_AAG | 8.39 (94.95) | -17.26 (188.80) | -11.84 (102.62) | -58.81 (209.37) | 0.56 (1.50) | 0.22 (2.87) |
| CAG_CAA | 34.31 (64.00) | 100.58 (115.35) | -9.70 (69.17) | 17.70 (129.49) | 0.70 (1.01) | 1.41 (1.75) |
| CAG_CAG | 29.33 (54.66) | -80.74 (109.23) | 52.46 (59.08) | -147.27 (121.39) | -0.36 (0.86) | 0.81 (1.66) |
| Minor diplotypes | -57.24 (78.46) | 181.55 (132.32) | -32.46 (84.80) | 144.14 (146.72) | -0.93 (1.24) | 1.04 (2.01) |
| CAG_AGG | REF | REF | REF | REF | REF | REF |
| p_interaction_‡ | 0.81 | | 0.67 | | 1.00 | |

* Comprised of SNPs as described in the Results section; †Models adjusted for sex, rs366631, *in utero* smoke exposure, current secondhand smoke exposure, current body mass index (BMI), and current height; ‡ Global F-test p-value: significance of interaction

Table S7a Adjusted estimates of diplotypes within *GSTM5* on lung function measurements at ages 10 years (n = 852) and 18 years (n = 716) by *in utero* smoke exposure status

|  | Forced expiratory volume in 1 second (FEV_1_) (mL) at age 10† | | Forced Vital Capacity  (FVC) (mL) at age 10 † | | FEV_1_/FVC (%) at age 10† | |
| --- | --- | --- | --- | --- | --- | --- |
| Diplotype* | Not exposed  (β, SE) | Exposed  (β, SE) | Not exposed  (β, SE) | Exposed  (β, SE) | Not exposed  (β, SE) | Exposed  (β, SE) |
| AA_AG | 30.16 (24.88) | 24.00 (44.03) | 15.42 (26.69) | -8.78 (42.91) | 0.85 (0.63) | 1.31 (1.19) |
| AA_CA | 66.45 (22.85) | 30.70 (44.53) | 52.74 (24.51) | -19.03 (43.40) | 0.89 (0.58) | 1.70 (1.20) |
| AG_CA | -30.02 (30.55) | 101.29 (74.18) | -25.56 (32.77) | 21.29 (72.30) | -0.25 (0.78) | 3.29 (2.00) |
| CA_CA | 7.99 (34.43) | -28.47 (58.55) | 14.74 (36.93) | -20.99 (57.06) | -0.05 (0.88) | -0.40 (1.58) |
| Minor diplotypes | -34.16 (46.36) | -25.45 (77.40) | -47.40 (49.72) | -38.22 (75.43) | -0.05 (1.18) | 0.31 (2.09) |
| AA_AA | REF | REF | REF | REF | REF | REF |
| p_interaction_‡ | 0.76 | | 0.88 | | 0.59 | |
|  | Forced expiratory volume in 1 second (FEV_1_) (mL) at age 18† | | Forced Vital Capacity  (FVC) (mL) at age 18 † | | FEV_1_/FVC (%) at age 18† | |
| Diplotype* | Not exposed  (β, SE) | Exposed  (β, SE) | Not exposed  (β, SE) | Exposed  (β, SE) | Not exposed  (β, SE) | Exposed  (β, SE) |
| AA_AG | 97.74 (55.47) | -31.70 (102.98) | 141.17 (60.64) | -37.24 (119.09) | -0.46 (0.85) | -0.55 (1.62) |
| AA_CA | -8.35 (51.05) | 3.63 (106.69) | 25.75 (55.81) | -128.36 (122.97) | -0.91 (0.79) | 1.60 (1.68) |
| AG_CA | -120.89 (70.34) | 34.71 (178.57) | 14.12 (76.90) | 60.84 (205.78) | -2.48 (1.08) | -0.61 (2.81) |
| CA_CA | 50.26 (77.03) | -82.96 (136.16) | 60.82 (84.22) | 67.98 (156.75) | -0.17 (1.18) | -2.77 (2.14) |
| Minor diplotypes | -8.59 (115.02) | -55.29 (332.13) | -76.04 (125.76) | -167.98 (381.99) | -0.56 (1.77) | 1.30 (5.23) |
| AA_AA | REF | REF | REF | REF | REF | REF |
| p_interaction_‡ | 0.65 | | 0.57 | | 0.47 | |

* Comprised of SNPs as described in the Results section; †Models adjusted for sex, rs366631, current smoking, current secondhand smoke exposure, current body mass index (BMI), and current height; additional adjustment for current smoking for lung function levels at age 18; ‡ Global F-test p-value: significance of interaction

Table S7b Adjusted estimates of diplotypes within *GSTM5* on lung function measurements at ages 10 years (n = 852) and 18 years (n = 716) by secondhand smoke exposure status

|  | Forced expiratory volume in 1 second (FEV_1_) (mL) at age 10† | | Forced Vital Capacity  (FVC) (mL) at age 10 † | | FEV_1_/FVC (%) at age 10† | |
| --- | --- | --- | --- | --- | --- | --- |
| Diplotype* | Not exposed  (β, SE) | Exposed  (β, SE) | Not exposed  (β, SE) | Exposed  (β, SE) | Not exposed  (β, SE) | Exposed  (β, SE) |
| AA_AG | 26.09 (31.46) | 32.64 (29.95) | 18.00 (33.09) | 2.40 (31.47) | 0.56 (0.81) | 1.41 (0.76) |
| AA_CA | 56.84 (28.96) | 62.54 (28.52) | 48.67 (30.47) | 25.70 (29.96) | 0.57 (0.75) | 1.67 (0.73) |
| AG_CA | -61.19 (39.93) | 27.39 (39.62) | -56.96 (42.01) | -1.00 (41.63) | -0.68 (1.03) | 1.44 (1.01) |
| CA_CA | -16.49 (45.04) | 23.90 (39.51) | 25.97 (47.38) | 7.58 (41.51) | -1.69 (1.16) | 1.02 (1.01) |
| Minor diplotypes | -17.78 (62.36) | -33.89 (52.07) | -70.32 (65.60) | -23.77 (54.71) | 1.51 (1.61) | -0.73 (1.33) |
| AA_AA | REF | REF | REF | REF | REF | REF |
| p_interaction_‡ | 0.83 | | 0.94 | | 0.26 | |
|  | Forced expiratory volume in 1 second (FEV_1_) (mL) at age 18† | | Forced Vital Capacity  (FVC) (mL) at age 18 † | | FEV_1_/FVC (%) at age 18† | |
| Diplotype* | Not exposed  (β, SE) | Exposed  (β, SE) | Not exposed  (β, SE) | Exposed  (β, SE) | Not exposed  (β, SE) | Exposed  (β, SE) |
| AA_AG | 186.53 (72.05) | -40.88 (67.02) | 138.62 (77.06) | 58.10 (75.94) | 1.22 (1.14) | -1.99 (1.01) |
| AA_CA | 30.69 (68.54) | -34.47 (62.83) | 48.07 (73.31) | -34.96 (71.08) | -0.63 (1.08) | -0.38 (0.95) |
| AG_CA | -65.87 (96.77) | -146.20 (89.30) | 7.77 (103.50) | 3.69 (101.05) | -1.81 (1.53) | -2.61 (1.34) |
| CA_CA | 3.25 (100.91) | 36.96 (90.46) | 62.74 (107.93) | 78.10 (102.31) | -1.44 (1.59) | -0.35 (1.36) |
| Minor diplotypes | -45.66 (147.08) | -65.45 (162.75) | -118.47 (157.31) | -20.57 (184.01) | 0.70 (2.32) | -0.87 (2.45) |
| AA_AA | REF | REF | REF | REF | REF | REF |
| p_interaction_‡ | 0.32 | | 0.95 | | 0.39 | |

* Comprised of SNPs as described in the Results section; †Models adjusted for sex, rs366631, *in utero* exposure, current body mass index (BMI), current height; additional adjustment for current smoking for lung function levels at age 18 ‡ Global F-test p-value: significance of interaction

Table S7c Adjusted estimates of diplotypes within *GSTM5* on lung function measurements at age 18 years by personal smoking status (n = 716)

|  | Forced expiratory volume in 1 second (FEV_1_) (mL) at age 18† | | Forced Vital Capacity  (FVC) (mL) at age 18 † | | FEV_1_/FVC (%) at age 18† | |
| --- | --- | --- | --- | --- | --- | --- |
| Diplotype* | Not exposed  (β, SE) | Exposed  (β, SE) | Not exposed  (β, SE) | Exposed  (β, SE) | Not exposed  (β, SE) | Exposed  (β, SE) |
| AA_AG | 126.02 (55.36) | -120.66 (100.88) | 131.13 (60.67) | 20.46 (113.95) | 0.17 (0.86) | -3.01 (1.55) |
| AA_CA | 63.71 (52.32) | -224.78 (94.70) | 63.21 (57.34) | -205.64 (106.39) | -0.08 (0.81) | -1.56 (1.45) |
| AG_CA | -169.34 (75.15) | 67.62 (129.22) | -36.21 (82.37) | 154.64 (145.44) | -2.84 (1.17) | -0.99 (1.98) |
| CA_CA | 72.64 (77.60) | -120.54 (131.21) | 145.94 (85.05) | -114.01 (147.35) | -1.16 (1.21) | -0.44 (2.01) |
| Minor diplotypes | -17.83 (116.34) | -276.13 (281.00) | -70.05 (127.52) | -76.56 (315.09) | 0.67 (1.81) | -4.49 (4.31) |
| AA_AA | REF | REF | REF | REF | REF | REF |
| p_interaction_‡ | **0.004** | | **0.05** | | 0.31 | |

* Comprised of SNPs as described in the Results section; †Models adjusted for sex, rs366631, *in utero* smoke exposure, current secondhand smoke exposure, current body mass index (BMI), and current height; ‡ Global F-test p-value: significance of interaction

Table S8a Adjusted estimates of diplotypes within *GSTM2* on repeated lung function measurements from ages 10 to 18 years by *in utero* smoke exposure status (1020 subjects, 1599 observations)

|  | Forced expiratory volume in 1 second (FEV_1_) (mL) † | | Forced Vital Capacity  (FVC) (mL) † | | FEV_1_/FVC (%)† | |
| --- | --- | --- | --- | --- | --- | --- |
| Diplotype* | Not exposed  (β, SE) | Exposed  (β, SE) | Not exposed  (β, SE) | Exposed  (β, SE) | Not exposed  (β, SE) | Exposed  (β, SE) |
| TC_AC | -7.31 (37.95) | -5.16 (57.76) | 5.76 (40.59) | -103.10 (55.16) | -0.33 (0.91) | 3.60 (1.63) |
| TC_TC | -35.56 (22.90) | 18.70 (39.16) | -42.47 (24.45) | -11.75 (37.50) | -0.07 (0.55) | 0.77 (1.07) |
| TG_AC | 5.53 (35.04) | -42.67 (58.50) | -4.72 (37.51) | -57.72 (56.01) | -0.10 (0.83) | 1.51 (1.59) |
| TG_TG | -3.06 (22.23) | 56.85 (38.80) | -8.93 (23.81) | 16.50 (37.10) | 0.13 (0.52) | 1.78 (1.07) |
| Minor diplotypes | -2.81 (127.07) | -- | -45.37 (136.40) | -- | 1.92 (2.88) | -- |
| TC_TG | REF | REF | REF | REF | REF | REF |
| p_interaction_‡ | 0.55 | | 0.52 | | 0.26 | |

* Comprised of SNPs as described in the Results section; †Models adjusted for sex, rs366631, current smoking, secondhand smoke exposure, body mass index (BMI), height and age; ‡ Global F-test p-value: significance of interaction; -- Not estimable

Table S8b Adjusted estimates of diplotypes within *GSTM2* on repeated lung function measurements from ages 10 to 18 years by secondhand smoke exposure status (1020 subjects, 1599 observations)

|  | Forced expiratory volume in 1 second (FEV_1_) (mL) † | | Forced Vital Capacity  (FVC) (mL) † | | FEV_1_/FVC (%)† | |  |
| --- | --- | --- | --- | --- | --- | --- | --- |
| Diplotype* | Not exposed  (β, SE) | Exposed  (β, SE) | Not exposed  (β, SE) | Exposed  (β, SE) | Not exposed  (β, SE) | Exposed  (β, SE) |  |
| TC_AC | 30.30 (48.68) | -11.90 (41.34) | 45.07 (51.73) | -58.36 (42.72) | -0.28 (1.13) | 1.56 (0.99) | |
| TC_TC | -46.00 (28.81) | -5.83 (26.83) | -47.60 (30.60) | -29.53 (27.68) | -0.06 (0.69) | 0.49 (0.62) | |
| TG_AC | -10.06 (43.32) | 24.15 (41.40) | -31.50 (46.06) | 18.44 (42.86) | 0.61 (1.02) | 0.37 (0.97) | |
| TG_TG | -2.88 (27.31) | 8.99 (26.80) | -6.13 (29.04) | -16.76 (27.78) | 0.32 (0.64) | 0.66 (0.62) | |
| Minor diplotypes | 186.75 (207.91) | 44.39 (151.88) | 13.48 (220.30) | 57.34 (157.10) | 6.50 (4.45) | -0.12 (3.43) | |
| TC_TG | REF | REF | REF | REF | REF | REF | |
| p_interaction_‡ | 0.92 | | 0.74 | | 0.45 | | |

* Comprised of SNPs as described in the Results section; †Models adjusted for sex, rs366631, current smoking, secondhand smoke exposure, body mass index (BMI), height and age; ‡ Global F-test p-value: significance of interaction

Table S8c Adjusted estimates of diplotypes within *GSTM2* on repeated lung function measurements from ages 10 to 18 years by personal smoking status (1020 subjects, 1599 observations)

|  | Forced expiratory volume in 1 second (FEV_1_) (mL) † | | Forced Vital Capacity  (FVC) (mL) † | | FEV_1_/FVC (%)† | |  |
| --- | --- | --- | --- | --- | --- | --- | --- |
| Diplotype* | Not exposed  (β, SE) | Exposed  (β, SE) | Not exposed  (β, SE) | Exposed  (β, SE) | Not exposed  (β, SE) | Exposed  (β, SE) |  |
| TC_AC | -0.34 (31.82) | 320.17 (176.51) | -19.60 (33.41) | -30.67 (193.00) | 0.49 (0.80) | 6.80 (2.55) | |
| TC_TC | -24.61 (19.84) | 76.36 (88.43) | -36.11 (20.80) | -88.48 (96.71) | 0.15 (0.50) | 0.47 (1.28) | |
| TG_AC | -1.54 (30.19) | -9.83 (174.76) | -12.31 (31.73) | -82.67 (199.50) | 0.37 (0.75) | 1.69 (2.52) | |
| TG_TG | 8.29 (19.36) | 43.39 (90.44) | -4.03 (20.34) | -19.68 (98.91) | 0.45 (0.48) | 2.07 (1.31) | |
| Minor diplotypes | 21.54 (124.41) | -- | -27.08 (131.10) | -- | 2.21 (2.93) | -- | |
| TC_TG | REF | REF | REF | REF | REF | REF | |
| p_interaction_‡ | 0.30 | | 0.75 | | 0.59 | | |

* Comprised of SNPs as described in the Results section; †Models adjusted for sex, rs366631, current smoking, secondhand smoke exposure, body mass index (BMI), height and age; ‡ Global F-test p-value: significance of interaction; -- Not estimable

Table S9a Adjusted estimates of diplotypes within *GSTM3* on repeated lung function measurements from ages 10 to 18 years by *in utero* smoke exposure status (1037 subjects, 1628 observations)

|  | Forced expiratory volume in 1 second (FEV_1_) (mL) † | | Forced Vital Capacity  (FVC) (mL) † | | FEV_1_/FVC (%)† | |  |
| --- | --- | --- | --- | --- | --- | --- | --- |
| Diplotype* | Not exposed  (β, SE) | Exposed  (β, SE) | Not exposed  (β, SE) | Exposed  (β, SE) | Not exposed  (β, SE) | Exposed  (β, SE) |  |
| AAA_AAA | -39.60 (33.04) | -71.57 (51.73) | -42.92 (35.40) | -79.78 (49.61) | 0.58 (0.79) | 0.73 (1.40) | |
| AGA_AAA | -66.43 (30.33) | -6.15 (57.30) | -87.15 (32.50) | -63.92 (54.92) | 0.69 (0.72) | 1.07 (1.53) | |
| AGA_GGG | -49.33 (25.83) | 9.73 (46.93) | -44.91 (27.68) | 7.47 (44.94) | 0.34 (0.61) | -0.05 (1.28) | |
| GGA_GGG | -33.48 (40.90) | -36.55 (62.74) | -54.05 (43.81) | -57.25 (60.11) | 1.03 (0.97) | 0.17 (1.74) | |
| GGG_GGG | -68.58 (25.68) | -38.21 (46.85) | -59.55 (27.52) | -25.50 (44.79) | 0.06 (0.61) | -0.10 (1.27) | |
| Minor diplotypes | -73.02 (30.32) | 20.20 (51.92) | -69.72 (32.33) | -0.31 (49.64) | -0.28 (0.73) | 0.10 (1.45) | |
| AAA_GGG | REF | REF | REF | REF | REF | REF | |
| p_interaction_‡ | 0.72 | | 0.87 | | 0.96 | | |

* Comprised of SNPs as described in the Results section; †Models adjusted for sex, rs366631, current smoking, secondhand smoke exposure, body mass index (BMI), height and age; ‡ Global F-test p-value: significance of interaction

Table S9b Adjusted estimates of diplotypes within *GSTM3* on repeated lung function measurements from ages 10 to 18 years by secondhand smoke exposure status (1037 subjects, 1628 observations)

|  | Forced expiratory volume in 1 second (FEV_1_) (mL) † | | Forced Vital Capacity  (FVC) (mL) † | | FEV_1_/FVC (%)† | |  |
| --- | --- | --- | --- | --- | --- | --- | --- |
| Diplotype* | Not exposed  (β, SE) | Exposed  (β, SE) | Not exposed  (β, SE) | Exposed  (β, SE) | Not exposed  (β, SE) | Exposed  (β, SE) |  |
| AAA_AAA | -81.38 (40.74) | -18.15 (37.58) | -82.81 (43.06) | -35.68 (39.12) | 0.13 (0.97) | 1.22 (0.86) | |
| AGA_AAA | -54.67 (39.74) | -52.44 (35.92) | -79.92 (42.05) | -86.21 (37.30) | 0.99 (0.93) | 0.73 (0.83) | |
| AGA_GGG | -32.03 (31.32) | -19.83 (32.11) | -51.86 (33.12) | -8.37 (33.41) | 1.51 (0.74) | -0.31 (0.73) | |
| GGA_GGG | -15.41 (49.99) | -26.85 (46.43) | -45.36 (52.92) | -51.27 (48.17) | 1.93 (1.17) | 0.35 (1.10) | |
| GGG_GGG | -74.52 (32.20) | -38.28 (30.97) | -81.42 (34.08) | -21.32 (32.17) | 0.78 (0.75) | -0.57 (0.72) | |
| Minor diplotypes | -91.14 (40.32) | -3.49 (34.54) | -91.14 (42.65) | -13.77 (35.62) | -0.37 (0.97) | 0.16 (0.82) | |
| AAA_GGG | REF | REF | REF | REF | REF | REF | |
| p_interaction_‡ | 0.41 | | 0.46 | | 0.06 | | |

* Comprised of SNPs as described in the Results section; †Models adjusted for sex, rs366631, current smoking, secondhand smoke exposure, body mass index (BMI), height and age; ‡ Global F-test p-value: significance of interaction

Table S9c Adjusted estimates of diplotypes within *GSTM3* on repeated lung function measurements from ages 10 to 18 years by personal smoking status (1037 subjects, 1628 observations)

|  | Forced expiratory volume in 1 second (FEV_1_) (mL) † | | Forced Vital Capacity  (FVC) (mL) † | | FEV_1_/FVC (%)† | |  |
| --- | --- | --- | --- | --- | --- | --- | --- |
| Diplotype* | Not exposed  (β, SE) | Exposed  (β, SE) | Not exposed  (β, SE) | Exposed  (β, SE) | Not exposed  (β, SE) | Exposed  (β, SE) |  |
| AAA_AAA | -47.02 (28.06) | -54.12 (122.77) | -48.61 (29.49) | -24.01 (135.70) | 0.48 (0.70) | 0.13 (0.19) | |
| AGA_AAA | -50.54 (26.89) | 71.15 (120.77) | -76.92 (28.27) | 83.79 (133.50) | 0.77 (0.67) | 0.18 (0.18) | |
| AGA_GGG | -37.56 (22.67) | 80.43 (111.08) | -33.32 (23.84) | 132.80 (123.20) | 0.22 (0.56) | 0.25 (0.17) | |
| GGA_GGG | -37.72 (34.41) | 47.88 (182.01) | -57.33 (36.16) | 103.50 (202.20) | 0.92 (0.86) | -0.11 (0.28) | |
| GGG_GGG | -62.01 (22.59) | 135.02 (103.36) | -49.49 (23.76) | 165.50 (114.60) | -0.27 (0.56) | 0.25 (0.16) | |
| Minor diplotypes | -49.53 (26.31) | 235.96 (128.51) | -50.70 (27.55) | 322.80 (142.70) | -0.10 (0.66) | -0.94 (0.20) | |
| AAA_GGG | REF | REF | REF | REF | REF | REF | |
| p_interaction_‡ | 0.28 | | 0.21 | | 0.82 | | |

* Comprised of SNPs as described in the Results section; †Models adjusted for sex, rs366631, current smoking, secondhand smoke exposure, body mass index (BMI), height and age; ‡ Global F-test p-value: significance of interaction

Table S10a Adjusted estimates of diplotypes within *GSTM4* on repeated lung function measurements from ages 10 to 18 years by *in utero* smoke exposure status (1028 subjects, 1616 observations)

|  | Forced expiratory volume in 1 second (FEV_1_) (mL) † | | Forced Vital Capacity  (FVC) (mL) † | | FEV_1_/FVC (%)† | |
| --- | --- | --- | --- | --- | --- | --- |
| Diplotype* | Not exposed  (β, SE) | Exposed  (β, SE) | Not exposed  (β, SE) | Exposed  (β, SE) | Not exposed  (β, SE) | Exposed  (β, SE) |
| AGG_AGG | -8.07 (28.55) | 22.38 (51.37) | 16.61 (30.43) | 64.16 (49.44) | -0.47 (0.67) | -0.73 (1.39) |
| CAA_AGG | 37.69 (30.23) | -19.18 (51.41) | 50.33 (32.22) | -11.77 (49.04) | -0.11 (0.71) | 0.0021 (1.41) |
| CAG_AAG | -7.33 (40.13) | -36.82 (80.18) | -11.57 (42.77) | -81.25 (77.19) | 0.20 (0.95) | 1.41 (2.16) |
| CAG_CAA | 12.33 (28.87) | 20.36 (47.29) | 47.04 (30.77) | 4.31 (45.02) | -0.97 (0.68) | 0.89 (1.30) |
| CAG_CAG | -10.43 (24.99) | -2.79 (40.39) | -0.83 (26.56) | 61.48 (38.59) | -0.26 (0.59) | -1.72 (1.10) |
| Minor diplotypes | 1.05 (32.26) | 19.49 (59.77) | 33.71 (34.38) | 34.13 (57.25) | -1.18 (0.76) | -0.11 (1.62) |
| CAG_AGG | REF | REF | REF | REF | REF | REF |
| p_interaction_‡ | 0.88 | | 0.46 | | 0.46 | |

* Comprised of SNPs as described in the Results section; †Models adjusted for sex, rs366631, current smoking, secondhand smoke exposure, body mass index (BMI), height and age; ‡ Global F-test p-value: significance of interaction

Table S10b Adjusted estimates of diplotypes within *GSTM4* on repeated lung function measurements from ages 10 to 18 years by secondhand smoke exposure status (1028 subjects, 1616 observations)

|  | Forced expiratory volume in 1 second (FEV_1_) (mL) † | | Forced Vital Capacity  (FVC) (mL) † | | FEV_1_/FVC (%)† | |  |
| --- | --- | --- | --- | --- | --- | --- | --- |
| Diplotype* | Not exposed  (β, SE) | Exposed  (β, SE) | Not exposed  (β, SE) | Exposed  (β, SE) | Not exposed  (β, SE) | Exposed  (β, SE) |  |
| AGG_AGG | -27.36 (35.78) | 12.12 (34.43) | -21.17 (37.58) | 56.19 (35.98) | 0.16 (0.84) | 1.02 (0.80) | |
| CAA_AGG | 35.62 (38.45) | 35.12 (35.34) | 78.20 (40.38) | 22.25 (36.88) | -0.85 (0.90) | 0.51 (0.83) | |
| CAG_AAG | -16.19 (53.33) | -3.68 (47.52) | -11.37 (56.08) | -24.19 (49.59) | -0.07 (1.22) | 0.61 (1.13) | |
| CAG_CAA | -25.25 (36.21) | 39.93 (33.09) | 13.60 (38.02) | 39.39 (34.57) | -1.02 (0.84) | 0.08 (0.77) | |
| CAG_CAG | -33.72 (31.32) | -2.06 (28.59) | 3.64 (32.89) | 14.68 (29.74) | -1.38 (0.73) | -0.31 (0.67) | |
| Minor diplotypes | -16.13 (40.04) | 15.77 (39.44) | 17.62 (42.01) | 35.87 (41.28) | -1.44 (0.94) | -0.62 (0.90) | |
| CAG_AGG | REF | REF | REF | REF | REF | REF | |
| p_interaction_‡ | 0.50 | | 0.36 | | 0.78 | | |

* Comprised of SNPs as described in the Results section; †Models adjusted for sex, rs366631, current smoking, secondhand smoke exposure, body mass index (BMI), height and age; ‡ Global F-test p-value: significance of interaction

Table S10c Adjusted estimates of diplotypes within *GSTM4* on repeated lung function measurements from ages 10 to 18 years by personal smoking status (1028 subjects, 1616 observations)

|  | Forced expiratory volume in 1 second (FEV_1_) (mL) † | | Forced Vital Capacity  (FVC) (mL) † | | FEV_1_/FVC (%)† | |  |
| --- | --- | --- | --- | --- | --- | --- | --- |
| Diplotype* | Not exposed  (β, SE) | Exposed  (β, SE) | Not exposed  (β, SE) | Exposed  (β, SE) | Not exposed  (β, SE) | Exposed  (β, SE) |  |
| AGG_AGG | -0.97 (24.94) | -93.97 (118.99) | 28.39 (26.19) | -54.05 (131.90) | -0.67 (0.62) | -0.57 (1.81) | |
| CAA_AGG | 21.48 (26.20) | -5.39 (117.08) | 36.72 (27.50) | -37.89 (129.80) | -0.21 (0.65) | 0.87 (1.78) | |
| CAG_AAG | -15.28 (35.78) | -17.26 (182.23) | -22.07 (37.57) | -58.81 (202.00) | 0.37 (0.88) | 0.22 (2.77) | |
| CAG_CAA | 16.48 (24.72) | 100.58 (111.33) | 39.24 (25.96) | 17.70 (125.00) | -0.59 (0.61) | 1.41 (1.69) | |
| CAG_CAG | -9.69 (21.32) | -80.74 (105.43) | 13.81 (22.34) | -147.30 (116.90) | -0.72 (0.53) | 0.81 (1.60) | |
| Minor diplotypes | 8.71 (28.46) | 181.55 (127.72) | 40.09 (29.88) | 144.10 (141.60) | -0.11 (0.71) | 1.04 (1.94) | |
| CAG_AGG | REF | REF | REF | REF | REF | REF | |
| p_interaction_‡ | 0.43 | | 0.42 | | 0.96 | | |

* Comprised of SNPs as described in the Results section; †Models adjusted for sex, rs366631, current smoking, secondhand smoke exposure, body mass index (BMI), height and age; ‡ Global F-test p-value: significance of interaction

Table S11a Adjusted estimates of diplotypes within *GSTM5* on repeated lung function measurements from ages 10 to 18 years by *in utero* smoke exposure status (1002 subjects, 1568 observations)

|  | Forced expiratory volume in 1 second (FEV_1_) (mL) † | | Forced Vital Capacity  (FVC) (mL) † | | FEV_1_/FVC (%)† | |
| --- | --- | --- | --- | --- | --- | --- |
| Diplotype* | Not exposed  (β, SE) | Exposed  (β, SE) | Not exposed  (β, SE) | Exposed  (β, SE) | Not exposed  (β, SE) | Exposed  (β, SE) |
| AA_AG | 27.45 (24.72) | 13.57 (42.68) | 16.94 (26.67) | -1.31 (4.08) | 0.39 (0.59) | 0.74 (1.08) |
| AA_CA | 58.03 (22.66) | 26.35 (42.97) | 43.66 (24.45) | -1.02 (4.13) | 0.37 (0.54) | 1.40 (1.07) |
| AG_CA | -39.49 (30.35) | 84.75 (71.36) | -34.39 (32.74) | 3.40 (6.88) | -0.85 (0.73) | 1.89 (1.77) |
| CA_CA | 2.80 (34.17) | -45.60 (56.53) | 12.12 (36.87) | -2.25 (5.42) | -0.25 (0.82) | -1.11 (1.41) |
| Minor diplotypes | -47.91 (46.26) | -30.53 (75.86) | -61.03 (49.85) | -2.98 (7.31) | -0.37 (1.11) | -0.01 (1.99) |
| AA_AA | REF | REF | REF | REF | REF | REF |
| p_interaction_‡ | 0.57 | | 0.69 | | 0.62 | |

* Comprised of SNPs as described in the Results section; †Models adjusted for sex, rs366631, current smoking, secondhand smoke exposure, body mass index (BMI), height and age; ‡ Global F-test p-value: significance of interaction

Table S11b Adjusted estimates of diplotypes within *GSTM5* on repeated lung function measurements from ages 10 to 18 years by secondhand smoke exposure status (1002 subjects, 1568 observations)

|  | Forced expiratory volume in 1 second (FEV_1_) (mL) † | | Forced Vital Capacity  (FVC) (mL) † | | FEV_1_/FVC (%)† | |  |
| --- | --- | --- | --- | --- | --- | --- | --- |
| Diplotype* | Not exposed  (β, SE) | Exposed  (β, SE) | Not exposed  (β, SE) | Exposed  (β, SE) | Not exposed  (β, SE) | Exposed  (β, SE) |  |
| AA_AG | 39.22 (30.82) | 29.90 (29.18) | 22.03 (32.61) | 5.54 (30.55) | 0.83 (0.72) | 0.40 (0.66) | |
| AA_CA | 59.33 (28.44) | 41.53 (27.66) | 57.63 (30.08) | 13.44 (28.99) | 0.01 (0.67) | 0.76 (0.62) | |
| AG_CA | -52.04 (39.57) | -2.77 (38.65) | -54.24 (41.88) | -18.45 (40.45) | -0.87 (0.95) | 0.20 (0.88) | |
| CA_CA | -29.96 (44.04) | 9.96 (38.65) | 9.82 (46.63) | 0.023 (40.42) | -1.88 (1.03) | 0.49 (0.88) | |
| Minor diplotypes | -21.15 (62.14) | -44.60 (52.10) | -82.36 (65.85) | -25.20 (54.26) | 1.27 (1.52) | -1.14 (1.25) | |
| AA_AA | REF | REF | REF | REF | REF | REF | |
| p_interaction_‡ | 0.74 | | 0.74 | | 0.08 | | |

* Comprised of SNPs as described in the Results section; †Models adjusted for sex, rs366631, current smoking, secondhand smoke exposure, body mass index (BMI), height and age; ‡ Global F-test p-value: significance of interaction

Table S11c Adjusted estimates of diplotypes within *GSTM5* on repeated lung function measurements from ages 10 to 18 years by personal smoking status (1002 subjects, 1568 observations)

|  | Forced expiratory volume in 1 second (FEV_1_) (mL) † | | Forced Vital Capacity  (FVC) (mL) † | | FEV_1_/FVC (%)† | |  |
| --- | --- | --- | --- | --- | --- | --- | --- |
| Diplotype* | Not exposed  (β, SE) | Exposed  (β, SE) | Not exposed  (β, SE) | Exposed  (β, SE) | Not exposed  (β, SE) | Exposed  (β, SE) |  |
| AA_AG | 23.13 (21.42) | -120.66 (97.48) | 6.66 (22.65) | 20.46 (110.10) | 0.69 (0.53) | -3.01 (1.50) | |
| AA_CA | 53.28 (20.08) | -224.78 (91.51) | 32.08 (21.23) | -205.60 (102.80) | 0.74 (0.49) | -1.57 (1.41) | |
| AG_CA | -21.68 (27.78) | 67.32 (124.87) | -29.06 (29.37) | 154.60 (140.50) | -0.26 (0.68) | -0.99 (1.92) | |
| CA_CA | -8.34 (29.24) | -120.54 (126.78) | 3.38 (30.92) | -114.00 (142.30) | -0.58 (0.72) | -0.44 (1.95) | |
| Minor diplotypes | -38.08 (39.63) | -276.13 (271.53) | -47.85 (41.88) | -76.56 (304.40) | -0.17 (0.99) | -4.49 (4.17) | |
| AA_AA | REF | REF | REF | REF | REF | REF | |
| p_interaction_‡ | 0.10 | | 0.34 | | 0.12 | | |

* Comprised of SNPs as described in the Results section; †Models adjusted for sex, rs366631, current smoking, secondhand smoke exposure, body mass index (BMI), height and age; ‡ Global F-test p-value: significance of interaction

Table S12 Significant associations between diplotypes within the *GSTM2-5* gene cluster and CpG site methylation levels by gene position

| Gene | n | Methylation site*,† | p-value‡ |
| --- | --- | --- | --- |
| *GSTM2*  (any diplotype) | 231 | cg06615940 | 0.01 |
|  | 231 | cg06970744 | 0.05 |
|  | 231 | cg07219542 | 0.04 |
| *GSTM3*  (any diplotype) | 231 | cg00297950 | <0.0001 |
|  | 231 | cg07050011 | 0.001 |
|  | 231 | cg10807101 | <0.0001 |
|  | 231 | cg23472215 | 0.003 |
|  | 231 | cg23645476 | <0.0001 |
| *GSTM4*  (any diplotype) | 230 | cg05793930 | 0.04 |
|  | 229 | cg11903880 | 0.03 |
|  | 230 | cg15069758 | 0.0002 |
|  | 230 | cg21451998 | <0.0001 |
|  | 229 | cg22247664 | <0.0001 |
| *GSTM5*  (any diplotype) | 225 | cg22864244 | <0.0001 |

*Name of methylation site is derived from Illumina ID

†14 out of 52 CpG sites in *GSTM2-5* presented significant associations

‡Wilcoxon rank-sum test p-value

Table S13 Adjusted general linear models examining the main effects of CpG sites within *GSTM2* cluster on lung function measurements at 18 years (n = 215)

|  | Forced expiratory volume in 1 second (FEV_1_) | | Forced Vital Capacity (FVC) | | FEV_1_/FVC | |
| --- | --- | --- | --- | --- | --- | --- |
| CpG site* | β (mL) | p-value | β (mL) | p-value | β (%) | p-value |
| cg06970744 | 4.43 | 0.39 | 6.75 | 0.25 | -.02 | 0.85 |

*Name of methylation site is derived from Illumina ID; †Models adjusted for sex, rs366631, *in utero* smoke exposure, secondhand smoke exposure, body mass index (BMI) and height

Table S14 Effects of *GSTM2* and *GSTM5* diplotypes on lung function levels at age 18

| Outcome | Factor | Intervening Variable | Total Effect | p-value | Direct Effect | p-value | Indirect Effect | p-value |
| --- | --- | --- | --- | --- | --- | --- | --- | --- |
| FEV_1_ at Age 18 | GSTM5 diplotype | cg06970744 | -0.03 | 0.62 | -0.03 | 0.61 | 0.0002 | 0.95 |
| FVC at Age 18 | GSTM5 diplotype | cg06970744 | 0.04 | 0.54 | 0.04 | 0.54 | 0.0003 | 0.95 |

Table S15 Cotinine levels within smoke exposure categories

| Smoke exposure categories |  | Cotinine levels (ng/mmol) | | p-value |
| --- | --- | --- | --- | --- |
|  | N (%) | Median | 5%, 95% |  |
| Passive smoke among nonsmokers  (n = 302) |  |  |  | <0.0001 |
| Yes | 120 (39.7) | 1.18 | 0.05, 7.89 |  |
| No | 182 (60.3) | 0.30 | 0.03, 25.77 |  |
| Active smoking* (n = 152) |  |  |  | 0.001 |
| Yes | 4 (2.6) | 86.65 | 7.52, 156.72 |  |
| No | 148 (97.4) | 0.23 | 0.02, 13.23 |  |

*Among those without secondhand smoke exposure

Table S16 Effect of active smoking on lung function levels at age 18

|  |  |  | Forced expiratory volume in 1 second (FEV_1_) | | Forced Vital Capacity (FVC) | | FEV_1_/FVC † | |
| --- | --- | --- | --- | --- | --- | --- | --- | --- |
|  | N | Active smoking | β (mL) | p-value§ | β (mL) | p-value§ | β (%) | p-value§ |
| Model 1* | 731 | Yes | -61.08 | 0.16 | 4.64 | 0.92 | -1.22 | 0.07 |
|  |  | No | REF | -- | REF | -- | REF | -- |
| Model 2† | 742 | Yes | -76.82 | 0.08 | -8.22 | 0.86 | -1.25 | 0.06 |
|  |  | No | REF | -- | REF | -- | REF | -- |
| Model 3‡ | 739 | Yes | -80.98 | 0.06 | -17.95 | 0.70 | -1.17 | 0.08 |
|  |  | No | REF | -- | REF | -- | REF | -- |
| Model 4§ | 716 | Yes | -82.58 | 0.06 | -7.31 | 0.88 | -1.44 | 0.03 |
|  |  | No | REF | -- | REF | -- | REF | -- |

* Models adjusted for *GSTM2* diplotype, sex, rs366631, current smoking, secondhand smoke exposure, body mass index (BMI), height and age

†Models adjusted for *GSTM3* diplotype, sex, rs366631, current smoking, secondhand smoke exposure, body mass index (BMI), height and age

†Models adjusted for *GSTM4* diplotype, sex, rs366631, current smoking, secondhand smoke exposure, body mass index (BMI), height and age

§Models adjusted for *GSTM5* diplotype, sex, rs366631, current smoking, secondhand smoke exposure, body mass index (BMI), height and age
